# Impact of the WHO Framework Convention on Tobacco Control on global cigarette consumption: quasi-experimental evaluations using interrupted time series analysis and in-sample forecast event modelling

**DOI:** 10.1136/bmj.l2287

**Published:** 2019-06-19

**Authors:** Steven J Hoffman, Mathieu J P Poirier, Susan Rogers Van Katwyk, Prativa Baral, Lathika Sritharan

**Affiliations:** 1 Global Strategy Lab, Dahdaleh Institute for Global Health Research, Faculty of Health and Osgoode Hall Law School, York University, 4700 Keele Street, Dahdaleh Building 2120, Toronto, Ontario, M3J 1P3 Canada; 2 Department of Global Health and Population, Harvard T H Chan School of Public Health, Harvard University, Boston, MA, USA; 3 Department of Health Research Methods, Evidence, and Impact and McMaster Health Forum, McMaster University, Hamilton, Canada; 4 School of Epidemiology and Public Health, Faculty of Medicine, University of Ottawa, Ottawa, Canada; 5 School of Kinesiology and Health Science, Faculty of Health, York University, Toronto, Canada

## Abstract

**Objective:**

To evaluate the impact of the WHO Framework Convention on Tobacco Control (FCTC) on global cigarette consumption.

**Design:**

Two quasi-experimental impact evaluations, using interrupted time series analysis (ITS) and in-sample forecast event modelling.

**Setting and population:**

71 countries for which verified national estimates of cigarette consumption from 1970 to 2015 were available, representing over 95% of the world’s cigarette consumption and 85% of the world’s population.

**Main outcome measures:**

The FCTC is an international treaty adopted in 2003 that aims to reduce harmful tobacco consumption and is legally binding on the 181 countries that have ratified it. Main outcomes were annual national estimates of cigarette consumption per adult from 71 countries since 1970, allowing global, regional, and country comparisons of consumption levels and trends before and after 2003, with counterfactual control groups modelled using pre-intervention linear time trends (for ITS) and in-sample forecasts (for event modelling).

**Results:**

No significant change was found in the rate at which global cigarette consumption had been decreasing after the FCTC’s adoption in 2003, using either ITS or event modelling. Results were robust after realigning data to the year FCTC negotiations commenced (1999), or to the year when the FCTC first became legally binding in each country. By contrast to global consumption, high income and European countries showed a decrease in annual consumption by over 1000 cigarettes per adult after 2003, whereas low and middle income and Asian countries showed an increased annual consumption by over 500 cigarettes per adult when compared with a counterfactual event model.

**Conclusions:**

This study finds no evidence to indicate that global progress in reducing cigarette consumption has been accelerated by the FCTC treaty mechanism. This null finding, combined with regional differences, should caution against complacency in the global tobacco control community, motivate greater implementation of proven tobacco control policies, encourage assertive responses to tobacco industry activities, and inform the design of more effective health treaties.

## Introduction

Tobacco consumption is one of the leading causes of preventable death worldwide. Each year, tobacco is responsible for about seven million deaths^1^ and for nearly US$500bn (£396bn; €449bn) worth of economic damage owing to excess healthcare system costs and lost productivity.^2^ It causes over 12% of premature deaths globally^3^ and incurs substantial social cost for smokers and non-smokers alike.^4^ Yet the global tobacco epidemic shows no signs of relenting. The World Health Organization predicts that the number of tobacco related deaths will increase to one billion in the 21st century—up from 100 million in the 20th century—without rapid implementation of global tobacco control measures.^2^ Yet one third of the world’s population is not protected by any of the six key priorities for tobacco control identified by WHO.^1^

**Fig 1:**
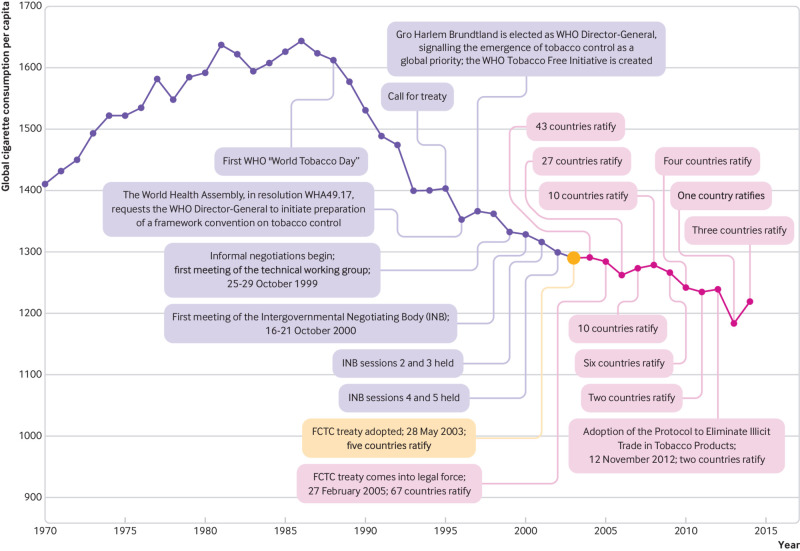
Timeline of major events in the creation, negotiation, adoption, and ratification of the WHO Framework Convention on Tobacco Control (FCTC)

The solution is often said to be the WHO Framework Convention on Tobacco Control (FCTC). Adopted under the auspices of WHO, this international treaty aims to reduce harmful tobacco consumption; lower smoking rates among children; and counteract the tobacco industry’s lobbying, advertising, and promotion activities. Treaty negotiations were called for in 1995, commenced in 1999 after the election of Gro Harlem Brundtland as WHO’s Director-General, and completed on 21 May 2003.^5^ The FCTC came into legal force on 27 February 2005 (fig 1). Fourteen years later, 181 countries have either ratified or acceded to the treaty, which means that all but 13 United Nations member states are legally bound by it. The FCTC is often put forward as a watershed moment in global health. For example, former WHO Director-General Margaret Chan said, “Without question, the WHO Framework Convention on Tobacco Control is the most powerful tool we have, as an international community, to reduce the global disease burden.”^6^

But has the FCTC actually reduced global cigarette consumption? Only a few attempts have been made so far to empirically evaluate the impact of this international treaty. National level studies have examined the impact of the treaty on domestic tobacco control policy and smoking prevalence in individual countries or regions.^7^ ^8^ ^9^ ^10^ ^11^ ^12^ ^13^ ^14^ ^15^ One recent large scale study has evaluated the aggregate impact of the national tobacco control policies that the FCTC recommends.^16^ These studies have shown that the individual policies promoted by the FCTC, including those prioritised in WHO’s MPOWER policy package (table 1),^17^ are effective at the national level when fully implemented.^18^ ^19^ ^22^

**Table 1:** WHO’s MPOWER policy package and representative studies that have shown the effectiveness of each tobacco control policy at the national level^7^ ^8^ ^9^ ^10^ ^11^ ^12^ ^13^ ^14^ ^16^ ^17^ ^18^ ^19^ ^20^ ^21^

MPOWER policy and description	Studies evaluating at national level (first author and year)
**Monitor tobacco use**	
Obtain nationally representative and population based periodic data on key indicators of tobacco use for youth and adults	Chung-Hall (2018); Katanoda (2014); Singh (2012)
**Protect people from tobacco smoke**	
Enact and enforce smoke free environments in healthcare and educational facilities as well as in all indoor public places including workplaces, restaurants and bars	Chung-Hall (2018); Gravely (2017); Katanoda (2014); Lunze (2012); Lv (2011); Martínez (2013); Sebrié (2012); Singh (2012); Thrasher (2008); Uang (2015)
**Offer help to quit tobacco use**	
Strengthen health systems so they can make tobacco cessation advice available as part of primary health care. Support quit lines and other community initiatives in conjunction with easily accessible, low cost pharmacological treatment where appropriate	Chung-Hall (2018); Gravely (2017); Katanoda (2014); Lunze (2012); Singh (2012)
**Warn about the dangers of tobacco**	
Require effective package warning labels	Chung-Hall (2018); Gravely (2017); Katanoda (2014); Lv (2011); Mir (2013); Singh (2012); Hiilamo (2015)
Implement counter-tobacco advertising	Chung-Hall et al. (2018); Hiilamo and Glantz (2017)
Obtain free media coverage of anti-tobacco activities	Chung-Hall (2018)
**Enforce bans on tobacco advertising, promotion, and sponsorship**	
Enact and enforce effective legislation that comprehensively bans any form of direct tobacco advertising, promotion, and sponsorship	Gravely (2017); Katanoda (2014); Lv (2011); Singh (2012)
Enact and enforce effective legislation to ban indirect tobacco advertising, promotion, and sponsorship	Gravely (2017); Katanoda (2014); Singh (2012)
**Raise taxes on tobacco products**	
Increase tax rates for tobacco products and ensure that they are adjusted periodically to keep pace with inflation and rise faster than consumer purchasing power	Chaloupka (2012); Chung-Hall (2018); Gravely (2017); Katanoda (2014); Lunze (2012); Singh (2012)
Strengthen tax administration to reduce the illicit trade in tobacco products	Chaloupka (2012); Chung-Hall (2018)

**Fig 2:**
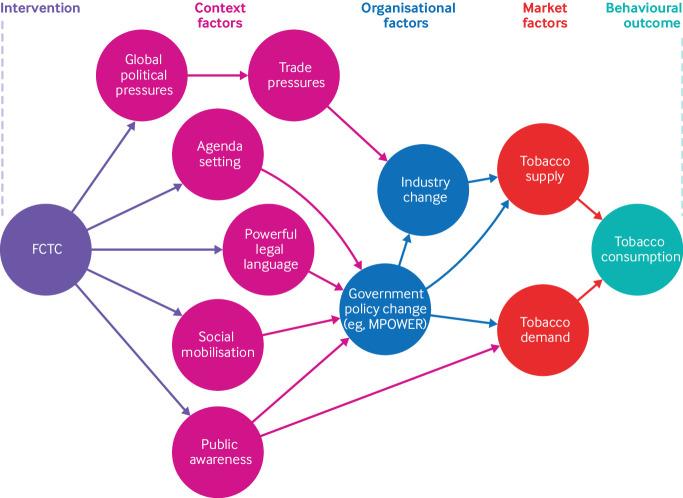
Logic model for conceptualising the impact of the WHO Framework Convention on Tobacco Control (FCTC) on tobacco consumption

What these studies do not address is whether international law—so often advocated as the solution to health challenges—is an effective tool for changing health behaviours.^23^ ^24^ ^25^ ^26^ ^27^ ^28^ Understanding the impact of the FCTC as an international legal instrument (distinct from understanding the efficacy of the FCTC’s specific recommendations on tobacco control) helps to determine whether an international treaty mechanism was necessary to address tobacco control and to inform whether international law should be used to manage other health challenges. The FCTC represents a culmination of political will to reduce the disease burden caused by tobacco, as well as the importance of tobacco control on the global health agenda,^29^ and we can thereby conceive of this international law as a global population health intervention that—through agenda setting, social mobilisation, public awareness, financial, trade, and social pressures, and powerful legal language—can result in local and national action to reduce tobacco consumption (fig 2). In other words, many tobacco control policies have been proven to be both efficacious and effective at the national level and some studies have shown the FCTC’s efficacy under ideal circumstances (that is, when the policies it promotes are fully implemented).^16^ ^18^ ^22^ However, no study so far has quasi-experimentally evaluated the effectiveness of the decision to adopt a tobacco control treaty as a strategy for reducing global cigarette consumption. 

International law is proposed, negotiated, adopted, ratified, and implemented in public political processes, making it impossible to conduct a randomised controlled trial or limit crossover effects between countries. Not only are there known capacity constraints in the ability of countries to implement international laws,^30^ there are also many routes in addition to direct legal obligations through which the intended outcomes of these international laws might take effect. For example, international laws can change global norms, empower transnational advocacy networks, and alter the expectations that countries have of each other, irrespective of whether they are legally binding on any one particular country. One could also expect market equilibrium effects, whereby action in one country will affect market dynamics in other countries. For example, strict regulations imposed by one government could encourage companies to move to jurisdictions with fewer rules to achieve greater profits.^31^ ^32^ In a globally interconnected and interdependent world, the effects of international laws are not limited to those who are formally parties to them.

Therefore, the best methodological approaches to measuring international laws’ effects are quasi-experimental impact evaluations.^33^ ^34^ Quasi-experimental research designs test descriptive causal hypotheses about manipulable causes to support a counterfactual inference about what would have happened in the absence of treatment, but lack the random assignment of units.^35^ Counterfactual inference can be reached using one of several approaches, such as a constant underlying time trend in the absence of an intervention (interrupted time series analysis) or a constant association between the outcome and the model’s explanatory variables before and after the intervention (in-sample forecast event modelling). Despite the strengths of interrupted time series analysis and in-sample forecast event modelling, these quasi-experimental approaches have never been used to evaluate an international law.^36^ ^37^ In fact, a systematic review of all quantitative impact evaluations of international laws found only one quasi-experiment—a difference-in-differences analysis of bilateral tax treaties’ impact on foreign investment^38^—despite these methods having been extensively used to evaluate laws, policies, and regulations in domestic contexts.^39^ ^40^ Another deficit has been the lack of high quality, internationally comparable data on tobacco consumption that is suitable for analysis by quasi-experiment. An open access dataset developed specifically for this purpose is now available.^41^ This dataset overcomes limitations of data that have been modelled with smoothing functions, such as those developed by the Institute for Health Metrics and Evaluation,^42^ which by definition lack breaks and discontinuities that are necessary to implement quasi-experimental approaches. Although no study can definitively claim to causally attribute a global discontinuity in cigarette consumption to the FCTC, these study designs are the best possible for establishing strong evidence of association in this context.

In this study, we sought to improve understanding of the global tobacco epidemic and to advance the way we understand and evaluate international laws more broadly. We took an exploratory approach to quasi-experimentally evaluate the FCTC’s impact on global cigarette consumption under different models, assumptions, and scenarios. Despite their limitations, large scale quantitative approaches allow for the incorporation of data from many more countries and time periods than would be practical with in-depth qualitative approaches.

## Methods

### Cigarette consumption data

This study used a previous systematic collection and quality appraisal of national cigarette consumption data from 1970 to 2015.^41^ In summary, an adaptive search strategy was used to collect data from all national statistical agencies on production, trade, and sales of cigarettes, supplemented with data from international sources, academic and grey literature, and subject matter experts. Academic databases were also used to identify research publications related to cigarette consumption, which were used to trace the source information or to contact researchers to request their data. Each country’s data were appraised by two researchers to evaluate intersource consistency and data confidence. This effort resulted in an open access dataset of national cigarette consumption estimates for 71 countries representing over 95% of the world’s cigarette consumption and 85% of the world’s population.^41^ Before this effort, the primary dataset of national cigarette consumption estimates made available to the public by the Institute for Health Metrics and Evaluation used imputed data and estimates that were synthesised, modelled, and smoothed using each country’s gross domestic product and regional dummy variables.^42^ This smoothing made the data, by definition, unsuitable for quasi-experimental impact evaluations that rely on breaks or discontinuities in the data.

### Definition of the intervention point

Theoretically, the FCTC could have achieved socialisation effects observed through the negotiating process (1999-2003), normative effects observed after the treaty’s adoption (2003), or legal effects observed through countries formally ratifying it (2005-present).^5^ Assuming the normative effect is dominant, the treaty’s adoption in 2003 was designated a priori as this study’s primary intervention point, meaning that the statistical analyses were designed to evaluate whether a discontinuity in cigarette consumption occurred from 2004 onwards. To ensure the robustness of any findings, we designated 1999 a priori as a secondary intervention point, which would test the potential socialisation effect of the FCTC’s negotiation and policy signalling in the lead-up to the treaty’s adoption in 2003. We also designated a priori the year when the FCTC became legally binding in each country as a secondary intervention point, which required centering (T_0_) each country’s cigarette consumption data on the year that country ratified or acceded to the FCTC. These robustness checks compared the time before the FCTC was binding on each country (T_−3_, T_−2_, T_−1_) with the time after the FCTC was binding (T_+1_, T_+2_, T_+3_; figure A2 in appendix 1) and would find any legal effects of the FCTC. Finally, while our primary focus on the FCTC’s overall real world effect is best evaluated with global consumption data, we also a priori designed stratified region and income level analyses to uncover any masked group effects and to identify causal mechanisms that might explain differences in cigarette consumption trends.

### Statistical analysis

Given the exploratory nature of this study, considerable efforts were undertaken to identify any potential impacts using different models, assumptions, and scenarios. We used two different statistical approaches to calculate whether observed changes were statistically significant discontinuities: interrupted time series analysis and in-sample forecast event modelling. The population weighted effects of the FCTC were also calculated for different groups of countries by UN region, country income level, and membership in the Organisation for Economic Co-operation and Development (OECD), and individually for the top 10 cigarette-consuming countries, in order to maximise the chances of finding effects.

Interrupted time series analysis has previously been used to evaluate the effects of different health policies^37^ and health related outcomes,^43^ including tobacco control policies^44^ and cigarette consumption.^45^ Our overall null hypothesis was that the FCTC was not associated with any changes in global population weighted cigarette consumption per capita for adults aged 15 years and older. We used interrupted time series analysis to detect any statistically significant changes in the level or slope of the rate of change in cigarette consumption per adult (that is, first differenced consumption) after the FCTC’s adoption in 2003. Time series data without any statistically significant discontinuities would prevent us from rejecting the null hypothesis. Alternatively, if such discontinuities existed, they could be attributable to a one-time change in consumption at the intervention point, or an interaction of consumption with time. Nearly all regional and country specific consumption patterns are non-linear and non-stationary, so we used first differencing to obtain annual changes in consumption, after which Dickey-Fuller tests confirmed (P<0.01) that all iterations were stationary (tables A2-3). First differencing refers to the use of year-over-year change in tobacco consumption as the unit of analysis rather than consumption itself, meaning that we are testing whether there has been a discontinuity in the rate of change of consumption (that is, an acceleration).

Event modelling is based on panel data time-series regression models with time varying coefficients. We constructed an event model based on a highly interacted series of annual country specific variables for 70 countries (not including Taiwan, owing to missing data) to obtain a predictive model of national cigarette consumption per adult selected using k-fold cross validation. This model rests on the assumption that a series of country specific variables relating to its economy, political system, tobacco industry, and human development (table A9) would be similarly correlated with cigarette consumption both before and after the FCTC’s adoption. In the absence of change, we would expect that forecasting consumption after 2003 based on in-sample correlations prior to 2003 between consumption and country specific variables (which are available for all years of analysis) would result in forecasted consumption estimates for the post-2003 period that are not statistically different than actual consumption. This in-sample forecasted counterfactual is represented by the following linear regression equation: *y_i,t_*=x*_i,t_*β+ε*_i,t_*; *i*=1,…,N; *t*=1,…*T* (where *y_i,t_* is cigarette consumption per adult, x*_i,t_* is a k-vector of independent variables theoretically expected to influence or predict cigarette consumption, β is a k-vector of coefficients, and ε*_i,t_* is the error term). This statistical test used the annual estimates of cigarette consumption per adult directly and did not require first differencing.

Further methodological notes and details of robustness checks performed for the interrupted time series analysis and event model are available in appendix 1. Our systematic effort to detect any potential FCTC effects included two quasi-experimental approaches at the global level, for subgroups by region, country income, OECD membership, and top cigarette-consuming country. We also conducted an additional 42 statistical analyses as robustness checks. Stata software codes used to implement all analyses are available in appendix 2.

### Patient and public involvement

Patients were not involved in this study. Findings will be actively disseminated through conference presentations, publications in academic journals, plain language policy notes, personalised briefings to leading global tobacco control organisations, and commentary in news media.

## Results

### Descriptive statistics

**Fig 3:**
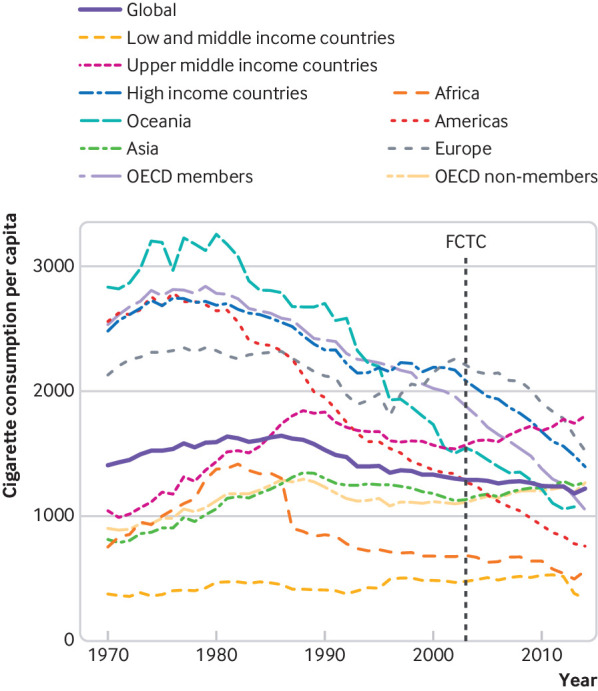
Annual population weighted data on cigarette consumption per capita, by country income level, UN region, and OECD membership status. Consumption based on number of cigarettes consumed per adult aged 15 years or older per year. FCTC=WHO Framework Convention on Tobacco Control, adopted in May 2003

We calculated annual, population weighted cigarette consumption per adult by UN region, country income level, and OECD membership in units of cigarettes consumed per adult per year (fig 3). As expected, global cigarette consumption decreased from around 1985. The quantities and trends of consumption according to different country groupings were highly variable, however, with high income countries and OECD member countries showing a particularly rapid decline in consumption. Upper middle income and Asian countries bucked the global trend of decreasing cigarette consumption over time, and continued to increase consumption rates to this day.

### Interrupted time series analysis

**Fig 4:**
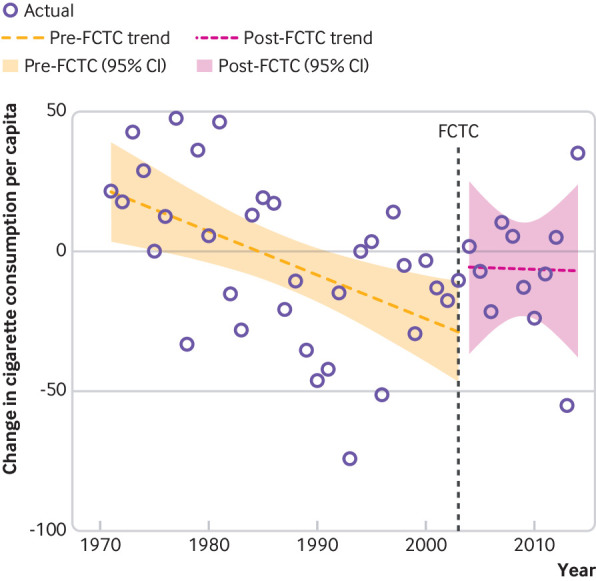
Interrupted time series plot of annual change in cigarette consumption per capita, with 95% confidence intervals, before and after 2003 adoption of the WHO Framework Convention on Tobacco Control (FCTC). Cigarette consumption data are based on first differencing (that is, use of year-over-year change in tobacco consumption as the unit of analysis rather than consumption itself) and weighted by global population. Consumption based on units of cigarettes consumed per adult aged 15 years or older per year

We saw no statistically significant change in either level or slope of change in global population weighted cigarette consumption per adult after 2003 (fig 4). Coefficients for both level and slope change for all units of analysis are presented in table 2. Owing to the use of first differencing in the interrupted time series analysis, level change coefficients represent a one-time decrease (negative coefficients) or increase (positive coefficients) of consumption per adult, and slope change coefficients represent the average rate of change of that acceleration or deceleration per year. Our results indicated that most regions and countries had no significant changes in their patterns in cigarette consumption per adult, with only Europe achieving a faster rate of decline after 2003. However, upper middle income countries, low and middle income countries, Oceania, the Americas, Asia, and China showed slower rates of decline after 2003, while India had a one-time increase in consumption rate but an accelerated decrease in consumption over time. Interrupted time series analysis for the secondary intervention points (that is, 1999 and realignment of country-year data according to when the FCTC came into legal force for each country) did not significantly affect these results (table A6 and figures A3-5). Global results for the interrupted time series analysis did not change after removal of China or countries that have divided since 1970 (Armenia, Azerbaijan, Belarus, Bosnia and Herzegovina, Croatia, Czechoslovakia, Czech Republic, Estonia, Kazakhstan, Moldova, Slovakia, Slovenia, Soviet Union, Ukraine, Uzbekistan, and Yugoslavia) from the sample.

**Table 2:** Results from interrupted time series analysis, with 2003 cutoff date for various subgroups

	Coefficient, standard error, 95% CI				Obs¶
	Underlying linear time trend	Level change at 2003*	Trend change starting 2003*	Constant	
All countries (n=71)	−1.67§, 0.42, −2.53 to −0.82	24.06†, 12.38, −0.95 to 49.08	1.73, 2.43, −3.18 to 6.65	22.48§, 7.567, 7.19 to 37.77	44
Income level					
High income (n=30)	−1.57, 0.96, −3.51 to 0.37	−22.68, 22.01, −67.16 to 21.81	0.31, 2.00, −3.72 to 4.35	14.74, 15.78, −17.16 to 46.65	44
Upper middle income (n=23)	−2.43‡, 0.99, −4.43 to −0.43	45.35‡, 18.79, 7.36 to 83.33	2.61, 3.06, −3.57 to 8.79	53.06‡, 21.86, 8.88 to 97.25	44
Lower middle income (n=13)	−0.12, 0.41, −0.96 to 0.72	10.19, 10.39, −10.85 to 31.23	−1.17, 1.09, −3.37 to 1.03	4.716, 7.059, −9.57 to 19.01	42
Low and middle income (n=41)	−1.63§, 0.58, −2.79 to −0.46	32.01‡, 14.88, 1.93 to 62.07	1.45, 3.51, −5.63 to 8.54	33.16‡, 13.24, 6.40 to 59.92	44
UN regions					
Africa (n=7)	−2.91§, 0.89, −4.72 to −1.11	43.51†, 25.72, −8.48 to 95.50	2.43, 3.79, −5.23 to 10.10	42.73†, 22.14, −2.00 to 87.47	44
Americas (n=9)	−2.40†, 1.20, −4.82 to 0.02	19.27, 21.14, −23.45 to 61.99	4.24‡, 1.98, 0.24 to 8.24	−0.727, 24.62, −50.48 to 49.03	44
Asia (n=24)	−1.86§, 0.58, −3.03 to −0.69	36.19§, 12.24, 11.46 to 60.92	1.28, 2.01, −2.79 to 5.34	38.60§, 13.28, 11.76 to 65.45	44
Europe (n=30)	0.11, 1.44, −2.79 to 3.02	−19.25, 38.43, −96.91 to 58.42	−8.72§, 2.99, −14.75 to −2.69	2.165, 16.55, −31.28 to 35.61	44
Oceania (n=1)	−5.37‡, 2.15, −9.72 to −1.03	109.6‡, 43.45, 21.73 to 197.5	1.68, 5.81, −10.07 to 13.43	41.78, 44.51, −48.25 to 131.8	43
Latin America (n=7)	−1.99, 1.15, −4.31 to −0.33	28.32, 26.18, −24.58 to 81.22	1.39, 2.88, −4.42 to 7.21	11.54, 21.95, −32.82 to 55.89	44
OECD membership					
OECD (n=28)	−2.91§, 0.79, −4.50 to −1.32	−2.18, 15.77, −34.05 to 29.70	1.48, 1.88, −2.32 to 5.28	27.21†, 16.16, −5.45 to 59.87	44
Non-OECD (n=43)	−1.34‡, 0.56, −2.47 to −0.20	29.21†, 15.50, −2.12 to 60.53	1.47, 3.20, −4.99 to 7.94	26.80‡, 11.25, 4.07 to 49.53	44
Excluding China (n=70)	−1.42‡, 0.65, −2.72 to −0.10	24.55†, 14.27, −4.30 to 53.40	−2.15, 2.07, −6.33 to 2.04	7.83, 11.84, −16.10 to 31.77	44
Countries that have not divided (n=57)	−1.66§, 0.41, −2.48 to −0.83	23.79, 12.01, −0.49 to 48.08	1.72†, 2.43, −3.19 to 6.63	22.22§, 7.56, 6.93 to 37.51	44
Top cigarette-consuming countries					
1. China	−2.51†, 1.37, −5.28 to 0.26	62.65§, 20.26, 21.69 to 103.6	1.58, 1.82, −2.09 to 5.25	68.39‡, 30.59, 6.58 to 130.2	44
2. Russia	−73.09‡, 29.25, −135.8 to −10.34	−9.38, 127.7, −283.4 to 264.6	56.88†, 31.44, −10.55 to 124.3	495.0§, 108.3, 262.8 to 727.3	18
3. USA	−3.25, 2.12, −7.54 to 1.03	22.55, 32.98, −44.11 to 89.20	4.89, 3.07, −1.31 to 11.08	2.55, 45.75, −89.91 to 95.00	44
4. Japan	−4.77§, 1.57, −7.95 to −1.60	−54.94, 36.78, −129.3 to 19.46	11.04†, 6.02, −1.13 to 23.20	75.78‡, 28.52, 18.10 to 133.5	43
5. Indonesia	−2.38, 1.43, −5.27 to 0.51	−23.43, 46.84, −118.3 to 71.40	19.44, 11.90, −4.64 to 43.52	57.35§, 19.95, 16.95 to 97.74	42
6. Philippines	−1.60, 3.78, −9.24 to 6.05	−16.77, 105.5, −230.4 to 196.9	26.46, 22.45, −18.99 to 71.90	6.53, 75.14, −145.6 to 158.6	42
7. India	−0.17, 0.21, −0.60 to 0.26	17.18‡, 6.604, 3.83 to 30.53	−1.51‡, 0.72, −2.96 to −0.05	−1.32, 3.85, −9.11 to 6.47	44
8. Brazil	−4.16‡, 1.85, −7.91 to −0.42	70.38, 47.69, −26.01 to 166.8	1.06, 3.65, −6.32 to 8.43	49.39, 31.44, −14.15 to 112.9	44
9. Turkey	−2.33, 3.39, −9.18 to 4.52	34.54, 86.84, −141.1 to 210.2	−24.50, 20.73, −66.42 to 17.43	53.30, 76.15, −100.7 to 207.3	43
10. Ukraine	24.91, 59.88, −103.5 to 153.3	−1.05, 207.3, −445.7 to 443.7	−69.62, 61.80, −202.2 to 62.92	95.05, 228.9, −395.9 to 586.0	18

* Positive (or negative) level change indicates a one-time increase (or decrease) in the rate of change of cigarette consumption per capita; positive (or negative) trend change indicates a continuing increase (or decrease) in the rate of change of cigarette consumption per capita after 2003.

† Coefficient at 90% confidence level.

‡ Coefficient at 95% confidence level.

§ Coefficient at 99% confidence level.

¶ Number of years used for each analysis (which differ depending on the data available for each region or country).

### In-sample forecast event model

**Fig 5:**
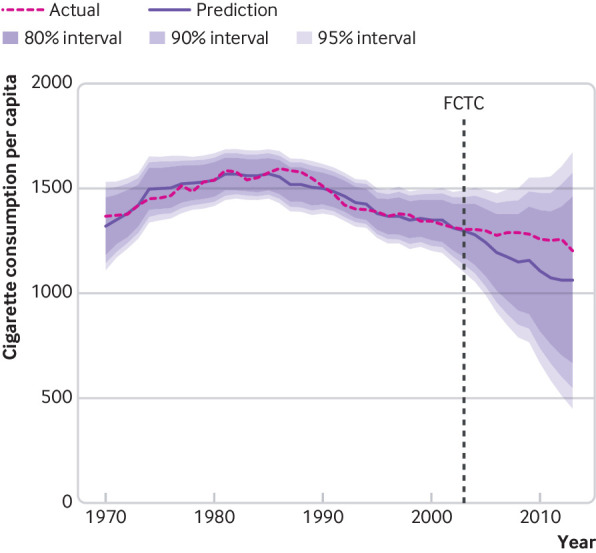
Population weighted, global event model predictions of cigarette consumption per capita, including 80%, 90%, and 95% prediction intervals, compared with actual consumption. In-sample forecast cutoff values begin in 2003 (adoption of the WHO Framework Convention on Tobacco Control (FCTC)), after which predictions are based on coefficients for the economy, political system, tobacco industry, and human development. Data are number of cigarettes consumed per adult aged 15 years or older per year

Event modelling indicated that cigarette consumption per adult did not decrease faster than would be expected following adoption of the FCTC in 2003. According to the event model (fig 5), the gap between predicted and actual consumption increased from five cigarettes per adult per year in 2003 to 150 cigarettes per adult per year in 2008. Although this finding would represent an increase in cigarette consumption over the modelled counterfactual, it was well within the 80%, 90%, and 95% prediction intervals (which would have required a difference of 238, 305, and 364 cigarettes per adult per year, respectively, to exceed them). Therefore, we cannot rule out the possibility that there was no difference, or that cigarette consumption had decreased compared with the counterfactual.

**Fig 6:**
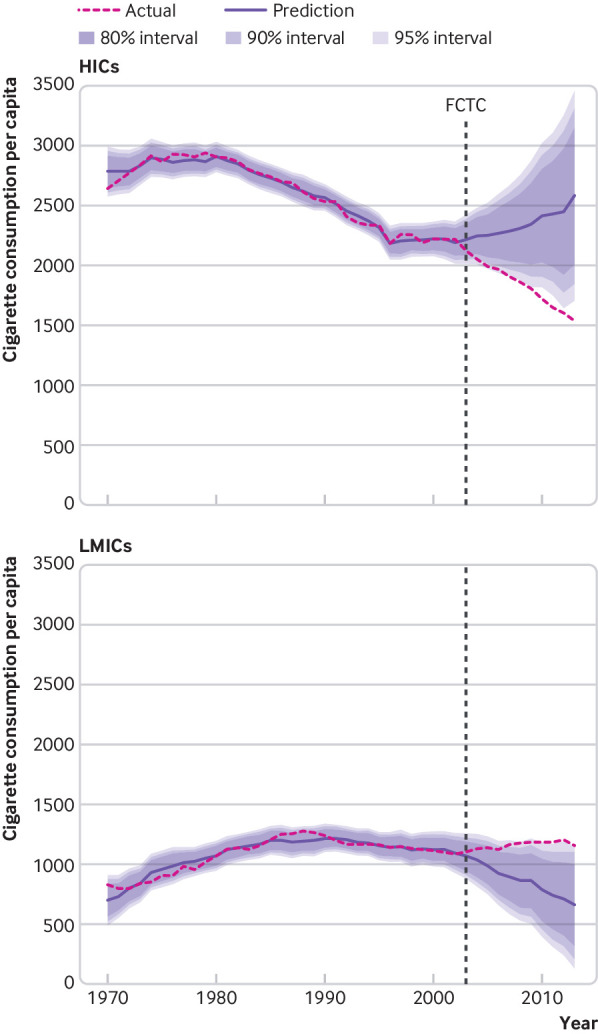
Population weighted, global event model predictions of cigarette consumption per capita for 29 high income countries (top panel) and 41 low and middle income countries (bottom panel), including 80%, 90%, and 95% prediction intervals, compared with actual consumption. In-sample forecast cutoff values begin in 2003 (adoption of the WHO Framework Convention on Tobacco Control (FCTC)), after which predictions are based on coefficients for the economy, political system, tobacco industry, and human development. Data are number of cigarettes consumed per adult aged 15 years or older per year

We also segmented the overall global event model by UN region and country income level. As figure 6 shows, cigarette consumption in high income countries fell below the 95% prediction interval by 2007, showing a reduction of over 1000 cigarettes per adult per year compared with the modelled counterfactual in 2013, 10 years after adoption of the FCTC. Data from low and middle income countries showed the opposite trend, with the average adult smoking over 500 cigarettes more per year than the modelled counterfactual predicted by 2013, rising above of the 95% prediction interval by 2010.

**Fig 7:**
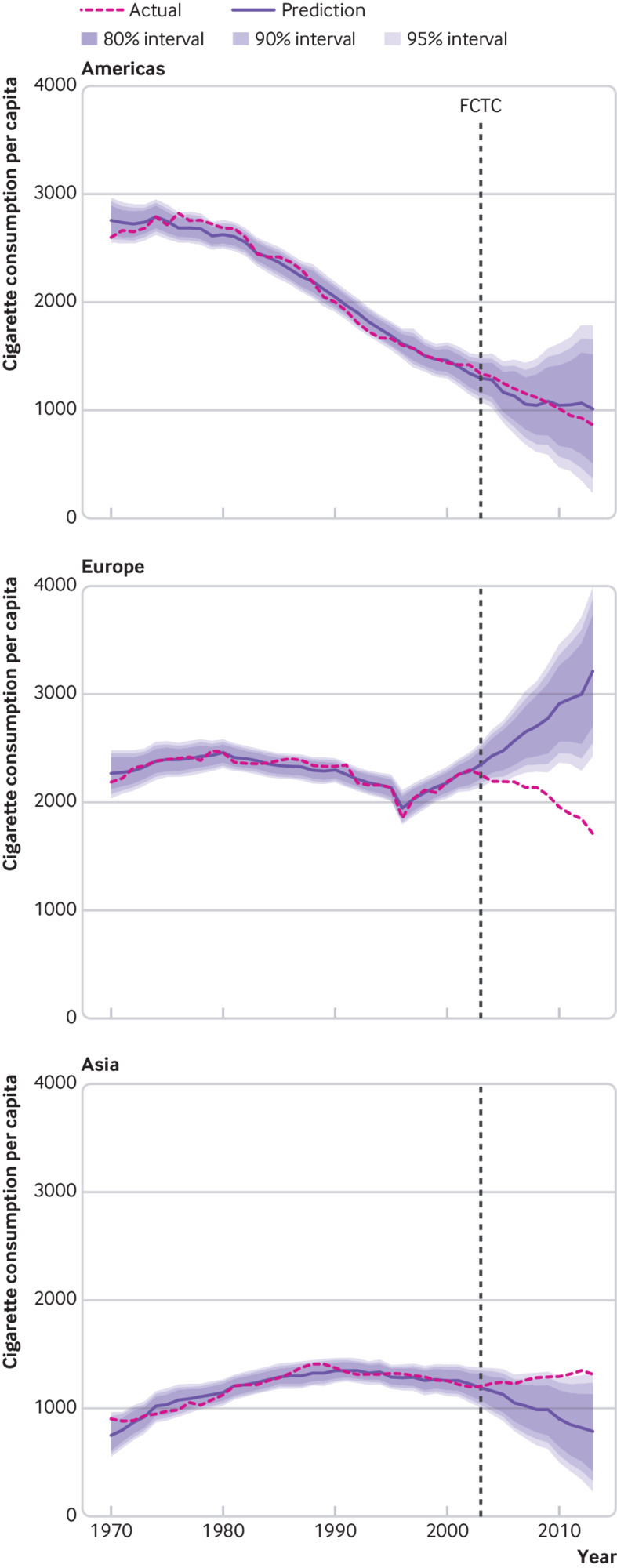
Population weighted, global event model predictions of cigarette consumption per capita for the Americas (top panel), Europe (middle panel), and Asia (bottom panel) with actual consumption. In-sample forecast cutoff values begin in 2003 (adoption of the WHO Framework Convention on Tobacco Control (FCTC)), after which predictions are based on coefficients for the economy, political system, tobacco industry, and human development. Data are number of cigarettes consumed per adult aged 15 years or older per year

We then grouped countries by UN region (fig 7), which reveals global patterns of cigarette consumption. The Americas (North, Central, and South) had already been on a downward consumption trend before 2003, and adoption of the FCTC appears to have done little to accelerate that trend, although separating the United States and Canada from the rest of the region (figure A8) echoes the divide in global income seen in figure 6. Conversely, European countries had been on an upward trend in consumption until a sudden reversal coinciding with the FCTC’s adoption, and Asian countries reversed a moderate downward trend in consumption at the same point in time. African consumption estimates were less certain, owing to a lack of verified data in most countries, but the available results also indicated actual consumption that was higher than the modelled counterfactual (figure A9). These results were robust to one and two year distributed lag models (figures A10-A11), the exclusion of China from global consumption, and the use of data that were not population weighted (figures A12-A13).

## Discussion

### Principal findings

After numerous statistical analyses, we could not find evidence that the rate at which global cigarette consumption per adult had been decreasing over the past three decades was accelerated by the adoption of the FCTC in 2003, whether through socialisation, normative, or legal pathways. Nevertheless, this null overall finding obscures a large discrepancy in trends after 2003, between high income countries and low and middle income countries, as well as between European and Asian countries. Although the interrupted time series analysis and event model did not allow us to reject the overall null hypothesis (that the FCTC did not accelerate reductions in global cigarette consumption per adult), the stratified analyses did uncover accelerated reductions in high income countries and European countries compared with predicted consumption based on trends before 2003. These stratified analyses also indicate that low and middle income countries and Asian countries have acutely reversed their previously decreasing consumption trends before 2003, to the point of consuming at least as many cigarettes as high income countries and European countries avoided in the period after FCTC adoption.

### Policy implications

The FCTC promotes evidence based tobacco control policies in nearly every country worldwide.^16^ ^20^ ^21^ ^46^ However, the empirical gap that we found between the FCTC’s efficacy and effectiveness accords with realities widely discussed in international legal scholarship, including cases of countries ignoring treaties after ratifying them, treaty provisions creating non-binding recommendations rather than binding obligations, insufficient governmental capacity to act on treaties, countries formally adopting treaty provisions into national policy without actual implementation, and multinational companies and illicit traders moving their activities to countries that have implemented fewer restrictions.^23^ ^24^ ^25^ ^26^ ^27^ ^28^ Indeed, these limitations of international law apply specifically to the FCTC, and could explain two apparent contradictions in this study’s findings.

The first apparent contradiction arises between the definitive evidence supporting the efficacy of key tobacco control policies^7^ ^8^ ^9^ ^10^ ^11^ ^12^ ^13^ ^14^ ^19^ and our finding that an international law promoting the adoption of these policies did not accelerate the global decline in cigarette consumption per adult. This conflict might be explained by the limited implementation of adopted tobacco control policies in low and middle income countries with lower governmental capacity,^18^ ^47^ by the absence of enforcement mechanisms in the FCTC motivating implementation,^26^ ^40^ by illicit trade in tobacco,^48^ and by the globalisation of harmful commercial determinants of health undermining global tobacco control efforts.^32^ The second apparent contradiction can be observed between simultaneously accelerated reductions in cigarette consumption in high income countries and European countries and newly increasing consumption in low and middle income countries and Asian countries after 2003. This conflict could be explained by European Union accession rules requiring stringent tobacco control measures among new members,^49^ and rapidly rising incomes resulting in greater affordability and demand for cigarettes in low and middle income countries.^47^ ^50^

By considering both paradoxical findings together, a compelling potential explanation of these findings emerges. Varied implementation of tobacco control policies and shifting trends in cigarette affordability across countries may have generated market equilibrium effects incentivising the tobacco industry to move its lobbying, marketing, and promotion activities away from high income countries (where they faced increasingly stringent regulations) and towards low and middle income countries and Asian countries (with far less stringent measures).^16^ ^22^ ^30^ ^31^ ^32^ If this is the case, the FCTC might even have unintentionally resulted in tobacco companies targeting people in low and middle income countries and Asian countries who would have fewer governmental protections against these companies’ efforts. Nevertheless, with the costs and consequences of the FCTC’s adoption now past, there is an urgent need for global strategies to rapidly scale the implementation of key tobacco control policies in low and middle income and Asian countries and to more assertively counteract the transnational activities of the tobacco industry.

### Strengths and limitations

Our quasi-experimental evaluations quantitatively assess the FCTC’s effects using a new open access dataset of national cigarette consumption estimates for 71 countries from verified data sources, covering 95% of global cigarette consumption and 85% of the world’s population.^41^ A strength of this study was the use of two complementary quasi-experimental approaches—interrupted time series analysis and event modelling—which both pointed to the same conclusion that global cigarette consumption trends have not changed substantially after the FCTC’s adoption in 2003. This conclusion was further reinforced by an extensive series of robustness checks presented in appendix 1.

This study was limited by the number of countries for which data were available, including limited availability of supply side data. The data did not include consumption of water tobacco, chewing tobacco, or loose leaf tobacco. Furthermore, cessation of tobacco use has a stronger protective effect on health than reduction in use, so aggregate consumption might not have fully captured the FCTC’s effects. Ten years might not have been long enough to observe the effects of the FCTC’s adoption, and low and middle income countries could increasingly benefit from measures such as legal defence against the tobacco industry’s use of international trade law to weaken tobacco control policies.^51^

The quasi-experimental methods implemented in this study have underlying assumptions. The interrupted time series analysis assumes a constant underlying time trend in the absence of an intervention, and the in-sample forecast event model assumes a constant association between cigarette consumption and the model’s explanatory variables before and after the intervention. Finally, we are unable to state with certainty that the associations observed are causal, owing to the exploratory nature of the study that purposefully included multiple testing under various models, assumptions, and scenarios. Quasi-experimental methods can be affected by omitted variable bias or confounding factors, which could lead to the masking of a true effect or finding a spurious association.

### Future research directions

Analysis of cigarette consumption trends has allowed us to discern patterns that could be useful in supporting future tobacco control efforts, including identifying countries to prioritise, the need for country specific strategies, and the importance of counteracting the tobacco industry. The divergence in cigarette consumption patterns between high income countries and low and middle income countries, and between European and Asian countries, should be studied in more detail. This emerging problem could continue to worsen owing to population growth, increasing living standards, and intensification of tobacco industry activities in more low and middle income countries and for a greater proportion of people within those countries. The degree to which governmental implementation capacity, market equilibrium effects, or any other factors have contributed to this difference should be identified and addressed in order to limit global tobacco use.

This research has demonstrated that more publicly available data are needed for the study of tobacco control. We could not have conducted quasi-experimental impact evaluations of the FCTC without a recently compiled dataset, which should have been part of treaty reporting requirements. Like all population health interventions, we should think critically about international laws and subject them to rigorous evaluation to determine whether their impact matches their rhetoric. Research should be conducted into the effects of the mechanisms underlying international law, the forums in which they are negotiated, and the parties involved in their negotiation to see how these factors might influence the ability of international laws to achieve their objectives.

**What is already known on this topic**

The World Health Organization’s Framework Convention on Tobacco Control (FCTC) has received nearly universal approval in academic literature, news media, and political speeches
The FCTC aims to reduce harmful tobacco consumption; lower smoking rates among children; and counteract the tobacco industry’s lobbying, advertising, and promotion activities
So far no studies have used a more rigorous quasi-experimental approach to evaluate the global impact of the FCTC to account for the fact that cigarette consumption had already been falling for at least 10 years before the international treaty was adopted in 2003

**What this study adds**

Using quasi-experimental approaches to evaluate the FCTC’s impact on global cigarette consumption per capita, this study showed no evidence of an acceleration in the global consumption rate (which had been decreasing over the past three decades) after adoption of the FCTC in 2003
High income and European countries showed accelerated decreases in consumption, whereas low and middle income and Asian countries showed increased consumption above what would have been anticipated without adoption of the FCTC
These results should motivate accelerated implementation of proven tobacco control policies in countries with uneven implementation and more assertive responses to transnational activities of the tobacco industry

## Web extra

Extra material supplied by authors

**Web appendix 1:** Appendix

**Web appendix 2:** FCTC Evaluation Stata Codes

## Notes

Contributors: SJH conceived the study, led its design as principal investigator, and supervised implementation. MJPP led the statistical analysis. All authors contributed to the study’s analysis and the preparation and writing of this manuscript, including reviewing, editing, and approving the final manuscript. SJH and MJPP are the guarantors. The corresponding author attests that all listed authors meet authorship criteria and that no others meeting the criteria have been omitted.

Ethical approval: This study was approved by McMaster University’s Hamilton integrated research ethics board (14-378). We attest that we have obtained appropriate permissions and paid any required fees for use of copyright protected materials.

Data sharing: The full dataset used in this study can be accessed at: https://dataverse.scholarsportal.info/dataverse/iccd. Additional data, methodological details, and code can be found in the appendices. All enquiries regarding the dataset and analyses can be made to the corresponding author.

The lead author (SJH) affirms that the manuscript is an honest, accurate, and transparent account of the study being reported; that no important aspects of the study have been omitted; and that any discrepancies from the study as originally planned have been explained.
