# Supplementary material for: Impact of the WHO Framework Convention on Tobacco Control on global cigarette consumption: quasi-experimental evaluations using interrupted time series analysis and in-sample forecast event modelling
Source: BMJ. 2019 Jun 19;365:l2287. doi: 10.1136/bmj.l2287 (PMC6582266; doi:10.1136/bmj.l2287)
Supplement: Supplementary file 1 — Web appendix 1: Appendix [file hofs048712.ww1.pdf]

APPENDIX

Appendix A. Methodological notes.

Data coverage is summarized in Figure A1. Over 80% of our sample population is covered by data until 2013, after which there is a slight drop before a sudden decrease to around 10% in 2015. Because of this, data from 2015 were excluded in both analyses. The source data was verified in order of net consumption,[1] so coverage of global consumption is likely to be near 95% for most years. All country-income levels used in analyses (Table A1) are from 2012 World Bank classifications using GNI per capita in US$ (Atlas methodology).[2] Our source data chose one year at which to divide data from countries that have split into constituent countries (USSR, Yugoslavia, etc.).[1] For the interrupted time-series analysis (ITS), all countries were included and a robustness check excluding these countries was run (Table 1). For the event analysis, only countries in existence in 2003 were included for analysis (i.e. Russia, Serbia and Montenegro, etc.) because a continuous trendline and data series were required for each in-sample forecast prediction. The FCTC signing and ratification-aligned sample used in the ITS has a lower consumption level (Figure A2) because non-ratifying countries (e.g. United States) were dropped from the sample.

We used the most common ITS approach of fitting least squares regression lines to both pre- and post- intervention data, which cannot be applied to non-linear or autocorrelated data.[3,4] Nearly all regional and country-specific consumption patterns are non-linear and non-stationary, so first differencing was used to obtain annual changes in consumption, after which Dickey-Fuller tests confirmed ($p<0.01$) that all iterations were stationary (Tables A2-3). First differencing refers to the use of year-over-year change in tobacco consumption as the unit of analysis rather than consumption itself, meaning that we are testing whether there has been a discontinuity in the rate of change of consumption – i.e., an acceleration. Durbin's alternative tests for serial correlation were then conducted, and all regions and countries displaying autocorrelation for lags 1 to 10 ($p<0.05$) were subjected to robustness checks (Tables A4-6). All pooled regional and global calculations were conducted on annual weighted averages of consumption by national population over the age of 15 using Stata command itsa.[5,6] Of the 21 series analyzed, only three demonstrated autocorrelation (United States, Philippines, and Turkey). After correcting for serial autocorrelation, Turkey's consumption change remains insignificant, while the United States and Philippines display a positive trend change (i.e., deceleration).

Finally, all country-specific ITS results for a 2003 intervention point are reported in Table A8. These results indicate that five countries experienced significant increases in consumption after the FCTC (Australia, Canada, China, Morocco, and South Africa), three countries had significant decreases in consumption (Denmark, Tunisia, and Serbia), two experienced mixed level and trend changes (Germany and India), and the remaining 58 countries had no significant change. Although these results are not representative of global changes in consumption, they support the general conclusions reached in the primary analysis.

For the event model analysis, some variables were not available for every country-year, so multiple imputation was used to fill any data gaps in order to obtain a strongly balanced panel. Years 2014-2015 were dropped from analysis due to low consumption data coverage. Five out of the 17 variables used (4.48% of all data) had missing data for at least one year - GDP, GDP growth, GDP per capita, trade index, and gender parity index. Chained multiple imputation with fifty imputations using linear regression (Stata command mi impute) was used to fill all data gaps.[7] Multiple-

Appendix to Hoffman SJ et al. 2019. BMJ. doi: 10.1136/bmj.l2287

imputation prediction (Stata command mi predictnl) was then used to calculate the predictive model, including 80%, 90%, and 95% prediction intervals[7], and mibeta was used to calculate adjusted and unadjusted R-squared coefficients.

Multiple event models were constructed and evaluated for goodness of fit using k-fold cross-validation. The independent variables used for modeling included primary tobacco company, log of GDP in current USD, annual GDP growth, log of GDP per capita at PPP, international trade as percentage of GDP, UNDP gender parity index, five V-Dem democracy indices of electoral democracy index, liberal democracy index, participatory democracy index, deliberative democracy index, and egalitarian democracy index, mean education of adults 25 and over for males and females, and education for women aged 15-44 (Table A9).[8–11] Models were built using country dummies, UN region dummies, UN subregion dummies, simplified (non-interacted) variables, a one-year distributed lag, a two-year distributed lag, only one GDP measure, and alternate time functional forms (Figure A8-A14). Once constructed, the models identified using k-fold cross-validation were then evaluated for global population-weighted per capita consumption root mean squared error (RMSE) for within model goodness of fit (pre-2004). The model with the lowest RMSE was identified as a fully interacted country-dummy model with no lagged explanatory variables (Table A10). Regardless of the model used, however, every model result in broadly consistent results. Country-specific models for the top ten tobacco consuming countries in 2010 (Figure A6) generally place actual consumption within the bounds of uncertainty for each in-sample forecast prediction, except for India and Brazil having higher than predicted consumption, and Russia having lower than predicted consumption. Results are robust to replacing multidimensional economic variables with a single measure of GDP (Figure A7).

References

1 Hoffman SJ, Mammone J, Katwyk SRV, *et al.* Comparable Cigarette Consumption Estimates for 71 Countries from 1970-2015: Systematic Collection of Data to Facilitate Quasi-Experimental Evaluations of National and Global Tobacco Control Interventions. *BMJ* 2019; **In Press**.

2 The World Bank. World Bank Country and Lending Groups – World Bank Data Help Desk. https://datahelpdesk.worldbank.org/knowledgebase/articles/906519-world-bank-country-and-lending-groups (accessed Oct 9, 2018).

3 Wagner AK, Soumerai SB, Zhang F, Ross-Degnan D. Segmented regression analysis of interrupted time series studies in medication use research. *J Clin Pharm Ther* 2002; **27**: 299–309.

4 Bernal JL, Cummins S, Gasparrini A. Interrupted time series regression for the evaluation of public health interventions: A tutorial. *Int J Epidemiol* 2017; **46**: 348–55.

5 Linden A. Conducting interrupted time-series analysis for single- and multiple-group comparisons. *Stata J* 2015; **15**: 480-.

6 Linden A. A comprehensive set of postestimation measures to enrich interrupted time-series analysis. *Stata J* 2017; **17**: 73–88.

7 White IR, Royston P, Wood AM. Multiple imputation using chained equations: Issues and guidance for practice. *Stat Med* 2011; **30**: 377–99.

8 Coppedge, Michael, Gerring J, Lindberg SI, *et al.* V-Dem Dataset v7. 2017.

9 Eriksen MP, Mackay J, Schluger NW, Islami F, Drope J. The tobacco atlas. Atlanta, Georgia: American Cancer Society, 2015.

10 The World Bank. World Bank Open Data. DataBank. https://data.worldbank.org/.

11 Institute for Health Metrics and Evaluation (IHME). Global Educational Attainment 1970-2015. Seattle, United States, 2015 http://ghdx.healthdata.org/record/global-educational-attainment-1970-2015.

Appendix Table 1. World Bank 2012 country-income classifications used in ITS and event model.[2]

	Country	Country-Income Level		Country	Country-Income Level
1	Algeria	Upper-Middle Income	36	Lebanon	Upper-Middle Income
2	Argentina	Upper-Middle Income	37	Lithuania	High Income
3	Armenia	Lower-Middle Income	38	Malaysia	Upper-Middle Income
4	Australia	High Income	39	Mexico	Upper-Middle Income
5	Austria	High Income	40	Moldova, Republic of	Lower-Middle Income
6	Azerbaijan	Upper-Middle Income	41	Morocco	Lower-Middle Income
7	Bangladesh	Low Income	42	Myanmar	Low Income
8	Belarus	Upper-Middle Income	43	Nepal	Low Income
9	Belgium	High Income	44	Netherlands	High Income
10	Bosnia and Herzegovina	Upper-Middle Income	45	Nigeria	Lower-Middle Income
11	Brazil	Upper-Middle Income	46	Pakistan	Lower-Middle Income
12	Bulgaria	Upper-Middle Income	47	Philippines	Lower-Middle Income
13	Canada	High Income	48	Poland	High Income
14	Chile	High Income	49	Portugal	High Income
15	China	Upper-Middle Income	50	Romania	Upper-Middle Income
16	Colombia	Upper-Middle Income	51	Russian Federation	High Income
17	Croatia	High Income	52	Saudi Arabia	High Income
18	Cuba	Upper-Middle Income	53	Serbia and Montenegro	Upper-Middle Income
19	Czech Republic	High Income	54	Slovakia	High Income
20	Denmark	High Income	55	Slovenia	High Income
21	Egypt	Lower-Middle Income	56	South Africa	Upper-Middle Income
22	Estonia	High Income	57	Spain	High Income
23	France	High Income	58	Sweden	High Income
24	Germany	High Income	59	Switzerland	High Income
25	Greece	High Income	60	Syrian Arab Republic	Lower-Middle Income
26	Hungary	Upper-Middle Income	61	Tanzania, United Republic of	Low Income
27	India	Lower-Middle Income	62	Thailand	Upper-Middle Income
28	Indonesia	Lower-Middle Income	63	Tunisia	Upper-Middle Income
29	Iran, Islamic Republic of	Upper-Middle Income	64	Turkey	Upper-Middle Income
30	Ireland	High Income	65	Ukraine	Lower-Middle Income
31	Italy	High Income	66	United Kingdom	High Income
32	Japan	High Income	67	United States	High Income
33	Kazakhstan	Upper-Middle Income	68	Uzbekistan	Lower-Middle Income
34	Korea, Democratic People's Republic of	Low Income	69	Venezuela, Bolivarian Republic of	Upper-Middle Income
35	Korea, Republic of	High Income	70	Viet Nam	Lower-Middle Income

Appendix Table 2. ITS results with 1999 cutoff for global, income-level country groupings, UN macro-regions, OECD membership, and top ten cigarette-consuming countries. Positive (negative) level change indicates a one-time increase (decrease) in the rate of change of cigarette consumption per capita, and positive (negative) trend change indicates a continuing increase (decrease) in the rate of change of cigarette consumption per capita in the post-1999 period.

	Time			Level Change 1999			Trend Change 1999			Constant			Obs.
	Coef.	SE	95% CI	Coef.	SE	95% CI	Coef.	SE	95% CI	Coef.	SE	95% CI	#
All countries	-2.078***	0.626	-3.345 - -0.812	17.67	14.08	-10.78 - 46.12	2.778*	1.579	-0.413 - 5.969	26.34***	8.391	9.380 - 43.30	44
High Income	-2.270*	1.319	-4.936 - 0.396	20.46	31.27	-42.73 - 83.66	-1.787	2.388	-6.613 - 3.039	21.47	17.19	-13.28 - 56.22	44
High Middle Income	-2.571*	1.304	-5.206 - 0.0633	10.27	20.84	-31.85 - 52.39	5.220**	2.262	0.648 - 9.791	54.43**	23.97	5.983 - 102.9	44
Low Middle Income	0.255	0.561	-0.882 - 1.391	-12.76	11.99	-37.04 - 11.51	0.592	1.026	-1.486 - 2.670	1.207	7.824	-14.63 - 17.05	42
Africa	-4.193***	1.258	-6.736 - -1.651	55.29*	30.41	-6.182 - 116.8	3.940	2.462	-1.035 - 8.916	54.89**	20.74	12.98 - 96.81	44
Oceania	-6.217**	2.839	-11.96 - -0.474	52.67	51.09	-50.67 - 156.0	8.849**	4.311	0.129 - 17.57	49.72	48.53	-48.44 - 147.9	43
Americas	-4.116***	1.392	-6.930 - -1.303	64.09***	21.68	20.28 - 107.9	3.259*	1.745	-0.268 - 6.786	15.53	24.83	-34.64 - 65.71	44
Asia	-1.545**	0.716	-2.991 - -0.0988	-7.388	13.06	-33.79 - 19.01	3.913**	1.468	0.946 - 6.879	35.65**	13.93	7.491 - 63.80	44
Europe	-1.425	2.219	-5.910 - 3.060	72.65	51.18	-30.79 - 176.1	-9.654***	3.411	-16.55 - -2.761	16.84	20.61	-24.82 - 58.51	44
OECD	-3.368***	0.971	-5.329 - -1.406	12.03	19.49	-27.36 - 51.42	0.955	1.751	-2.584 - 4.494	31.77*	16.94	-2.462 - 66.00	44
Non-OECD	-1.650**	0.809	-3.284 - -0.0159	16.65	16.44	-16.57 - 49.88	2.910	1.997	-1.126 - 6.946	29.75**	12.71	4.056 - 55.45	44
China	-2.201	1.783	-5.805 - 1.403	-2.216	26.33	-55.43 - 51.00	6.044***	2.214	1.568 - 10.52	65.47*	33.01	-1.257 - 132.2	44
Russia	-319	0	-319 - -319	229.3	0	229.3 - 229.3	292.5	0	292.5 - 292.5	634	0	634 - 634	18
USA	-4.344*	2.563	-9.524 - 0.836	30.74	41.09	-52.30 - 113.8	5.469	3.668	-1.945 - 12.88	13.39	48.68	-84.98 - 111.8	44
Japan	-4.614*	2.350	-9.368 - 0.140	-37.54	43.97	-126.5 - 51.40	4.625	4.639	-4.758 - 14.01	74.32**	33.49	6.574 - 142.1	43
Indonesia	-0.0900	1.487	-3.100 - 2.920	-128.5**	50.74	-231.3 - -25.80	15.25*	7.618	-0.177 - 30.67	35.39*	18.90	-2.865 - 73.65	42
Philippines	-2.463	5.335	-13.26 - 8.338	-15.38	99.47	-216.7 - 186.0	17.93	15.19	-12.83 - 48.68	14.96	84.35	-155.8 - 185.7	42
India	0.0495	0.243	-0.442 - 0.541	-1.159	7.189	-15.69 - 13.37	0.109	0.603	-1.109 - 1.328	-3.419	3.957	-11.42 - 4.578	44
Brazil	-7.645***	1.841	-11.37 - -3.924	168.1***	50.33	66.34 - 269.8	0.907	3.810	-6.794 - 8.608	81.43***	29.42	21.96 - 140.9	44
Turkey	-0.992	4.243	-9.574 - 7.591	6.588	84.06	-163.4 - 176.6	-16.24	12.83	-42.20 - 9.708	40.03	81.54	-124.9 - 205.0	43
Ukraine	-315	0	-315 - -315	421.9	0	421.9 - 421.9	291.6	0	291.6 - 291.6	409	0	409 - 409	18

Appendix Table 3. Dickey-Fuller test of stationarity for global, income-level country groupings, UN regions, OECD membership, and top ten cigarette-consuming countries after first-differencing.

	N	Zt	p
Global	43	-5.761	0.000
High Income	43	-4.359	0.000
High Middle Income	43	-5.821	0.000
Low Middle Income	43	-5.031	0.000
Africa	43	-5.295	0.000
Oceania	42	-6.480	0.000
Americas	43	-5.980	0.000
Asia	43	-5.228	0.000
Europe	43	-5.106	0.000
OECD	43	-4.215	0.000
Non-OECD	43	-5.945	0.000
China	43	-5.247	0.000
Russia	17	-2.946	0.005
USA	43	-8.826	0.000
Japan	42	-4.204	0.000
Indonesia	41	-5.662	0.000
Philippines	41	-10.192	0.000
India	43	-5.043	0.000
Brazil	43	-4.821	0.000
Turkey	42	-8.110	0.000
Ukraine	17	-2.434	0.014

Appendix Table 4. Durbin's alternative tests for serial correlation for global, income-level country groupings, UN regions, OECD membership, and top ten cigarette-consuming countries for first 10 lags after first-differencing for both 2003 and 1999 cutoff years.[1]

	N	p (lag 1)	p (lag 2)	p (lag 3)	p (lag 4)	p (lag 5)	p (lag 6)	p (lag 7)	p (lag 8)	p (lag 9)	p (lag 10)
Global 2003	44	0.213	0.467	0.350	0.490	0.615	0.653	0.774	0.799	0.725	0.743
Global 1999	44	0.104	0.222	0.302	0.470	0.421	0.357	0.451	0.506	0.604	0.712
High Income 2003	44	0.243	0.436	0.145	0.259	0.387	0.524	0.659	0.436	0.440	0.562
High Income 1999	44	0.327	0.590	0.283	0.446	0.572	0.704	0.812	0.549	0.573	0.682
High Middle Income 2003	44	0.624	0.435	0.564	0.712	0.681	0.494	0.630	0.743	0.731	0.796
High Middle Income 1999	44	0.685	0.507	0.608	0.765	0.720	0.509	0.645	0.751	0.755	0.760
Low Middle Income 2003	42	0.988	0.999	0.914	0.957	0.921	0.652	0.698	0.690	0.692	0.321
Low Middle Income 1999	42	0.948	0.996	0.810	0.838	0.896	0.718	0.782	0.819	0.881	0.848
Africa 2003	44	0.432	0.629	0.823	0.893	0.955	0.974	0.962	0.680	0.786	0.828
Africa 1999	44	0.617	0.834	0.949	0.981	0.992	0.997	0.981	0.591	0.694	0.672
Oceania 2003	43	0.128	0.095	0.189	0.189	0.300	0.256	0.345	0.377	0.394	0.419
Oceania 1999	43	0.131	0.057	0.133	0.083	0.154	0.183	0.272	0.361	0.429	0.436
Americas 2003	44	0.970	0.177	0.194	0.324	0.106	0.168	0.129	0.130	0.204	0.281
Americas 1999	44	0.482	0.415	0.551	0.644	0.398	0.499	0.445	0.434	0.546	0.662
Asia 2003	44	0.855	0.894	0.721	0.802	0.877	0.718	0.125	0.198	0.057	0.100
Asia 1999	44	0.924	0.937	0.739	0.769	0.833	0.635	0.120	0.178	0.031*	0.055
Europe 2003	44	0.920	0.915	0.086	0.117	0.189	0.247	0.305	0.426	0.531	0.441
Europe 1999	44	0.488	0.112	0.060	0.108	0.166	0.077	0.051	0.072	0.103	0.052
OECD 2003	44	0.800	0.857	0.345	0.426	0.262	0.377	0.512	0.576	0.509	0.496
OECD 1999	44	0.672	0.865	0.411	0.499	0.322	0.431	0.567	0.635	0.564	0.574
Non-OECD 2003	44	0.446	0.526	0.741	0.716	0.810	0.612	0.724	0.634	0.706	0.699
Non-OECD 1999	44	0.404	0.627	0.818	0.811	0.848	0.692	0.791	0.740	0.833	0.863
China 2003	44	0.429	0.221	0.399	0.566	0.498	0.482	0.460	0.589	0.071	0.076
China 1999	44	0.326	0.184	0.347	0.517	0.491	0.447	0.454	0.580	0.028*	0.008**
Russia 2003	18	0.231	0.056	0.091	0.186	0.341	0.483	0.513	0.564	0.180	0.304
Russia 1999	18	0.430	0.540	0.667	0.837	0.855	0.931	0.963	0.989	0.976	0.969
USA 2003	44	0.011*	0.042*	0.056	0.084	0.039*	0.018*	0.032*	0.019*	0.030*	0.037*
USA 1999	44	0.006**	0.024*	0.040*	0.050*	0.036*	0.015*	0.030*	0.022*	0.033*	0.047*
Japan 2003	43	0.784	0.305	0.453	0.541	0.697	0.763	0.837	0.896	0.937	0.950

[1] Values at significant at the 95% level marked with *; significant at 99% level with **.

Japan 1999	43	0.651	0.334	0.447	0.585	0.737	0.823	0.868	0.866	0.924	0.905
Indonesia 2003	42	0.700	0.779	0.765	0.865	0.415	0.473	0.473	0.513	0.590	0.706
Indonesia 1999	42	0.483	0.319	0.293	0.450	0.336	0.469	0.441	0.459	0.333	0.397
Philippines 2003	42	0.000**	0.000**	0.000**	0.000**	0.000**	0.000**	0.000**	0.000**	0.000**	0.000**
Philippines 1999	42	0.000**	0.000**	0.000**	0.000**	0.000**	0.000**	0.000**	0.000**	0.000**	0.000**
India 2003	44	0.406	0.330	0.436	0.112	0.169	0.178	0.117	0.176	0.176	0.212
India 1999	44	0.142	0.180	0.341	0.042*	0.077	0.125	0.092	0.077	0.043*	0.077
Brazil 2003	44	0.222	0.433	0.558	0.730	0.843	0.417	0.134	0.196	0.234	0.291
Brazil 1999	44	0.469	0.503	0.474	0.631	0.768	0.091	0.006**	0.006**	0.001**	0.001**
Turkey 2003	43	0.003**	0.011*	0.030*	0.063	0.121	0.174	0.175	0.258	0.368	0.488
Turkey 1999	43	0.003**	0.009**	0.024*	0.054	0.104	0.153	0.146	0.226	0.330	0.440
Ukraine 2003	18	0.431	0.212	0.348	0.460	0.399	0.551	0.668	0.141	0.143	0.063
Ukraine 1999	18	0.180	0.378	0.613	0.705	0.764	0.764	0.883	0.237	0.445	0.525

Appendix Table 5. Robustness checks for lagged ITS results with 2003 cutoff for all iterations with significant Durbin's alternative test for serial correlation. Positive (negative) level change indicates a one-time increase (decrease) in the rate of change of cigarette consumption per capita, and positive (negative) trend change indicates a continuing increase (decrease) in the rate of change of cigarette consumption per capita in the post-2003 period.

	AR Model	Time			Level Change			Trend Change			Constant			Obs.
	# of lags	Coef.	SE	95% CI	Coef.	SE	95% CI	Coef.	SE	95% CI	Coef.	SE	95% CI	#
USA	10	-3.253**	1.553	-6.392 - -0.113	22.55	29.49	-37.05 - 82.15	4.889**	1.971	0.906 - 8.873	2.545	29.65	-57.38 - 62.47	44
Philippines	10	-1.595	1.194	-4.012 - 0.823	-16.77	35.80	-89.24 - 55.69	26.46***	6.001	14.31 - 38.60	6.528	24.58	-43.23 - 56.29	42
Turkey	3	-2.332	2.418	-7.222 - 2.559	34.54	64.70	-96.32 - 165.4	-24.50*	13.65	-52.11 - 3.115	53.30	49.37	-46.56 - 153.1	43

Appendix to Hoffman SJ et al. 2019. BMJ. doi: 10.1136/bmj.l2287

Appendix Table 6. Robustness checks for lagged ITS results with 1999 cutoff for all iterations with significant Durbin's alternative test for serial correlation. Positive (negative) level change indicates a one-time increase (decrease) in the rate of change of cigarette consumption per capita, and positive (negative) trend change indicates a continuing increase (decrease) in the rate of change of cigarette consumption per capita in the post-1999 period.

	AR Model	Time			Level Change			Trend Change			Constant			Obs.
	# of lags	Coef.	SE	95% CI	Coef.	SE	95% CI	Coef.	SE	95% CI	Coef.	SE	95% CI	#
Asia	9	-1.545**	0.661	-2.882 - -0.208	-7.388	11.37	-30.37 - 15.59	3.913***	1.196	1.495 - 6.331	35.65***	12.32	10.74 - 60.55	44
China	10	-2.201	2.129	-6.503 - 2.101	-2.216	31.37	-65.62 - 61.19	6.044*	3.019	-0.0572 - 12.14	65.47*	36.46	-8.229 - 139.2	44
USA	10	-4.344**	1.828	-8.039 - -0.649	30.74	37.84	-45.74 - 107.2	5.469***	1.986	1.456 - 9.482	13.39	27.09	-41.36 - 68.15	44
Philippines	10	-2.463*	1.449	-5.396 - 0.471	-15.38	30.06	-76.23 - 45.47	17.93***	4.271	9.280 - 26.57	14.96	25.20	-36.06 - 65.97	42
India	4	0.0495	0.211	-0.378 - 0.477	-1.159	7.974	-17.28 - 14.96	0.109	0.692	-1.290 - 1.508	-3.419	4.093	-11.69 - 4.853	44
Brazil	10	-7.645***	1.067	-9.801 - -5.490	168.1***	29.43	108.6 - 227.5	0.907	2.664	-4.478 - 6.292	81.43***	15.12	50.86 - 112.0	44
Turkey	3	-0.992	2.845	-6.746 - 4.762	6.588	64.84	-124.6 - 137.7	-16.24*	8.663	-33.77 - 1.278	40.03	54.14	-69.47 - 149.5	43

Appendix to Hoffman SJ et al. 2019. BMJ. doi: 10.1136/bmj.l2287

Appendix Table 7. ITS results for global consumption aligned by actual country-year, with countries aligned by year FCTC signed, and with countries aligned by year FCTC ratified. Positive (negative) level change indicates a one-time increase (decrease) in the rate of change of cigarette consumption per capita, and positive (negative) trend change indicates a continuing increase (decrease) in the rate of change of cigarette consumption per capita in the post-intervention period.

	Time			Level Change			Trend Change			Constant			Obs.
	Coef.	SE	95% CI	Coef.	SE	95% CI	Coef.	SE	95% CI	Coef.	SE	95% CI	#
Actual Year[2]	-1.393***	(0.434)	-2.271 - -0.515	24.634**	(10.092)	4.222 - 45.046	-0.972	(1.772)	-4.556 - 2.613	24.595**	(9.359)	5.665 - 43.524	43
Actual Year (2 lags)	-1.393***	(0.380)	-2.162 - -0.624	24.634**	(10.303)	3.795 - 45.474	-0.972	(1.069)	-3.135 - 1.191	24.595***	(6.893)	10.651 - 38.538	43
Year Signed	-1.614***	(-2.445)	-2.445 - -0.783	24.411**	(4.467)	4.467 - 44.354	-0.778	(-3.111)	-3.111 - 1.554	26.449***	(8.916)	8.916 - 43.982	41
Year Ratified	-1.450***	(-2.307)	-2.307 - -0.592	15.798	(-7.807)	-7.807 - 39.403	-0.977	(-6.450)	-6.45 - 4.497	26.912***	(8.155)	8.155 - 45.669	42
Year Ratified (2 lags)	-1.450***	(0.331)	-2.118 - -0.782	15.798	(11.518)	-7.519 - 39.115	-0.977	(1.516)	-4.046 - 2.092	26.912***	(5.925)	14.918 - 38.906	42

[2] Values at significant at the 95% level marked with **; significant at 99% level with ***

Appendix Table 8. ITS results for every country with sufficient data coverage for a 2003 intervention point. Positive (negative) level change indicates a one-time increase (decrease) in the rate of change of cigarette consumption per capita, and positive (negative) trend change indicates a continuing increase (decrease) in the rate of change of cigarette consumption per capita in the post-intervention period.

	Time trend	SE	Level change (2003)	SE	Trend Change	SE	Constant	SE	Observations
Algeria	4.841	(7.369)	-8.894	(65.50)	-12.50	(8.366)	-105.1	(122.1)	34
Azerbaijan	28.15	(129.3)	207.3	(572.2)	-51.79	(133.9)	-128.7	(388.9)	18
Argentina	-2.180	(1.838)	76.62	(51.75)	-3.241	(5.856)	20.44	(49.60)	44
Australia	-5.371**	(2.147)	109.6**	(43.47)	1.677	(5.818)	41.73	(44.50)	43
Austria	-0.913	(1.984)	-61.52	(56.17)	7.548	(4.962)	12.97	(29.09)	44
Bangladesh	0.0474	(0.434)	8.080	(14.07)	-1.302	(4.118)	-2.677	(7.936)	41
Armenia	-21.36	(82.74)	-46.04	(303.7)	-7.599	(87.12)	284.0	(337.8)	17
Belgium	0.942	(2.303)	-50.02	(43.35)	1.733	(7.408)	-37.11	(45.90)	44
Bosnia and Herzegovina	19.61	(51.27)	-242.6	(518.1)	-56.20	(67.66)	161.6	(134.7)	21
Brazil	-4.164**	(1.850)	70.12	(47.71)	1.094	(3.653)	49.41	(31.44)	44
Bulgaria	0.344	(7.110)	-174.6	(338.3)	-3.371	(37.38)	51.70	(142.0)	44
Burma/Myanmar	1.468	(2.042)	-52.39	(35.81)	6.210	(5.054)	7.219	(9.870)	24
Belarus	-32.31	(69.20)	187.5	(330.3)	54.12	(77.72)	14.22	(203.6)	16
Canada	-3.827	(2.935)	28.98	(72.07)	12.01**	(4.847)	3.159	(43.01)	44
Chile	-0.427	(2.055)	-44.86	(55.91)	7.555	(8.769)	9.088	(46.43)	44
China	-2.514*	(1.373)	62.83***	(20.27)	1.566	(1.814)	68.38**	(30.65)	44
Croatia	1.825	(18.28)	115.1	(164.6)	-14.42	(21.40)	-103.5	(114.5)	23
Cuba	6.089	(9.539)	-2.941	(77.64)	-6.890	(10.62)	-129.0	(160.7)	29
Czech Republic	-46.77	(31.65)	316.2*	(181.2)	26.18	(32.77)	218.3	(152.4)	21
Denmark	-0.574	(1.158)	93.36*	(51.23)	-21.08**	(8.881)	7.519	(26.09)	44
Estonia	30.04	(19.73)	-118.0	(368.8)	-36.08	(33.24)	-64.49	(49.09)	18
France	-2.913***	(0.877)	-81.90	(68.92)	15.64*	(8.721)	39.71**	(17.64)	44
Germany	-0.837	(1.708)	-170.4***	(55.52)	21.06***	(6.369)	6.205	(33.80)	44
Greece	-2.016	(3.859)	44.07	(143.3)	-31.87	(22.53)	68.47	(50.12)	44
Hungary	-6.916	(4.170)	125.1	(205.7)	-12.26	(25.09)	88.51	(67.10)	44
India	-0.168	(0.211)	17.45**	(6.571)	-1.545**	(0.712)	-1.304	(3.861)	44

Indonesia	-2.386	(1.430)	-23.40	(46.81)	19.46	(11.87)	57.38***	(19.99)	42
Iran, Islamic Rep.	-2.228	(3.305)	91.64	(136.7)	-16.41	(21.64)	27.36	(66.79)	36
Ireland	-0.484	(2.277)	-143.8	(86.73)	11.46	(9.876)	-15.92	(46.86)	44
Italy	-1.552	(1.313)	-36.28	(34.71)	1.189	(4.452)	35.13	(24.59)	44
Japan	-4.774***	(1.567)	-54.89	(36.77)	11.05*	(6.025)	75.78**	(28.48)	43
Kazakhstan	-1.685	(75.30)	41.35	(207.5)	-13.39	(77.83)	67.58	(276.9)	17
South Korea	-4.858	(2.972)	27.29	(191.8)	7.732	(21.43)	83.34*	(47.44)	44
Lebanon	149.9	(380.0)	-419.0	(788.6)	-126.2	(382.9)	-551.2	(1,261)	17
Lithuania	-147.6*	(74.07)	433.2	(366.4)	155.1*	(78.25)	360.0	(238.0)	18
Malaysia	-2.361	(5.678)	58.50	(201.8)	-9.164	(20.41)	27.22	(66.55)	44
Mexico	-0.552	(1.026)	-16.97	(39.08)	3.307	(6.853)	-15.39	(21.42)	44
Morocco	-2.823**	(1.206)	51.16**	(24.51)	-0.988	(3.156)	47.00*	(26.95)	43
Nepal	-1.140	(0.784)	43.38	(31.11)	-1.180	(3.865)	27.71*	(14.06)	43
Netherlands	-0.872	(2.957)	-16.35	(64.09)	3.028	(11.17)	-8.352	(74.59)	44
Nigeria	-1.290	(1.147)	28.47	(19.83)	-1.624	(2.901)	11.62	(10.60)	26
Pakistan	0.856	(5.684)	-10.73	(60.40)	-3.248	(7.563)	5.320	(31.36)	24
Philippines	-1.598	(3.769)	96.27	(120.4)	-10.11	(19.13)	6.569	(74.96)	44
Poland	-6.744	(4.687)	76.10	(98.86)	0.0578	(6.245)	88.57	(79.14)	44
Portugal	-2.186	(1.875)	-51.06	(142.4)	1.006	(14.95)	53.77*	(28.21)	44
Romania	0.119	(4.107)	-0.0917	(145.4)	-15.64	(16.01)	9.312	(35.34)	44
Saudi Arabia	-2.867	(14.43)	183.3	(286.4)	-4.165	(18.48)	14.07	(247.3)	44
Slovakia	-14.43	(33.03)	31.06	(216.5)	23.29	(38.67)	-4.497	(176.1)	20
Democratic Republic of Vietnam	1.277	(1.859)	-23.10	(63.26)	-2.912	(6.858)	19.87	(24.50)	39
Slovenia	10.23	(11.30)	-37.20	(124.5)	-27.09	(17.14)	-38.00	(88.10)	23
South Africa	-4.641***	(1.052)	70.89***	(16.85)	4.693**	(2.162)	58.09**	(25.24)	44
Spain	-1.901	(2.259)	7.609	(67.00)	-15.56	(9.909)	42.47	(40.13)	44
Sweden	-1.754	(3.069)	12.11	(61.99)	3.537	(5.132)	9.479	(56.19)	44
Switzerland	-0.444	(3.530)	-34.87	(66.16)	-0.0683	(6.888)	-26.24	(74.80)	44
Syria	-3.763	(4.060)	-16.86	(133.2)	42.21	(42.06)	78.48	(97.15)	41
Thailand	-2.287	(1.411)	59.17	(48.45)	-1.250	(4.919)	34.45	(23.57)	44
Tunisia	-1.815	(2.291)	83.04	(65.41)	-20.25**	(8.230)	48.31	(35.44)	43
Turkey	-2.331	(3.388)	34.55	(86.82)	-24.49	(20.72)	53.28	(76.15)	43

Appendix to Hoffman SJ et al. 2019. BMJ. doi: 10.1136/bmj.l2287

Ukraine	24.93	(59.86)	-0.930	(207.3)	-69.64	(61.78)	94.92	(228.8)	18
Russia	-73.02**	(29.24)	-9.657	(128.0)	56.80*	(31.44)	494.9***	(108.1)	18
Egypt, Arab Rep.	-2.750	(1.860)	10.66	(57.98)	-3.297	(8.861)	65.52	(41.03)	43
United Kingdom	-2.229	(5.223)	35.99	(118.1)	1.038	(5.516)	-1.580	(59.31)	44
Tanzania	-0.509	(1.351)	33.63	(23.40)	-1.472	(2.803)	1.185	(29.52)	43
United States	-3.256	(2.118)	22.56	(32.99)	4.902	(3.069)	2.597	(45.75)	44
Uzbekistan	-5.771	(32.42)	66.44	(166.8)	7.725	(33.17)	-53.59	(65.30)	17
Venezuela, RB	-4.249	(2.548)	122.4	(79.80)	-5.767	(11.39)	34.14	(53.93)	43
Serbia	77.83**	(29.22)	-287.4	(272.9)	-140.3***	(33.95)	-337.8*	(190.6)	23

Appendix to Hoffman SJ et al. 2019. BMJ. doi: 10.1136/bmj.l2287

Appendix Table 9. Summary of variables used in the construction of the event model including sample mean, standard deviation, range, variable type, rationale for inclusion, and source.

Variable	Mean	Std. Dev.	Range	Variable type	Rationale	Source
Year	N/A	N/A	1970 to 2013	Interval	Year used as continuous variable in order to conduct in-sample forecast.	N/A
Country dummy	N/A	N/A	All countries	Categorical	Used to construct country-dummy model.	N/A
Primary tobacco company	N/A	N/A	China National Tobacco Corp (2%); Philip Morris International (37%) British American Tobacco (24%); Imperial Tobacco Group (3%); Japan Tobacco International (9%); Other (24%)	Categorical	The primary tobacco company present in the country could alter the market dynamics.	Tobacco Atlas[9]
Log GDP (Current $US)	25.27	1.76	18.23 to 30.45	Interval	Captures the size of the overall economy (i.e. the size of large economies might make the market behave differently, regardless of whether the country is wealthy or not).	World Bank[10]
Annual GDP growth (%)	4.04	4.95	-21.6 to 89.0	Interval	Captures how well the overall economy is doing (i.e. an economy that is growing at 5% will likely affect cigarette consumption differently than one experiencing a 2% contraction).	World Bank[10]
Log GDP Per Capita (Current $PPP)	8.88	1.08	4.96 to 11.48	Interval	This captures the relative wealth of the average person in a country (i.e. citizens of Norway are richer than Americans even though their economy is smaller).	World Bank[10]
Trade Openness (%)	63.43	35.71	-44.62 to 220.40	Interval	Trade openness measured as the sum of exports and imports of goods and services as a share of gross domestic product. Note that sums can exceed 100% if exports and imports are valued higher than national GDP. Captures the effect of trade and globalization on tobacco consumption.	World Bank[10]
Gender Parity Index	0.95	0.11	0.17 to 1.27	Interval	Gender parity index for gross enrollment ratio in primary education is the ratio of girls to boys enrolled at primary level in public and private schools. Captures the changing influence of gender inequality on tobacco consumption	World Bank[10]
Polyarchy Index	0.55	0.31	0.01 to 0.95	Interval	A composite index of freedom of association, clean elections, freedom of expression, elected officials, and suffrage variables capturing an ideal electoral democracy	V-Dem[8]
Liberal Democracy Index	0.45	0.31	0.01 to 0.92	Interval	A composite index of limits on political power, civil liberties, independent judiciary, and effective checks and balances capturing an ideal liberal democracy	V-Dem[8]

Participatory Democracy Index	0.35	0.23	0.01 to 0.81	Interval	A composite index of engagement in civil society, direct democracy, and subnational elected bodies capturing an ideal participatory democracy	V-Dem[8]
Deliberative Democracy Index	0.44	0.32	0.00 to 0.91	Interval	A composite index of the processes by which decisions are reached in a polity capturing an ideal deliberative democracy	V-Dem[8]
Egalitarian Democracy Index	0.45	0.28	0.04 to 0.89	Interval	A composite index of protecting the rights and freedoms, distributing resources equally, and enjoying equal access to power for all social groups capturing an ideal egalitarian democracy	V-Dem[8]
Mean education M+F, 25+ (Years)	7.48	3.38	0.93 to 14.69	Interval	Mean years of education achieved by both men and women aged 25 and over. Captures the effect of overall population education level on tobacco consumption.	IHME[11]
Mean education M, 25+ (Years)	7.95	3.13	1.48 to 14.59	Interval	Mean years of education achieved by men aged 25 and over. Captures one half of the gender-based inequality in population education level achievement on tobacco consumption.	IHME[11]
Mean education F, 25+ (Years)	7.00	3.66	0.27 to 14.79	Interval	Mean years of education achieved by women aged 25 and over. Captures one half of the gender-based inequality in population education level achievement on tobacco consumption.	IHME[11]
Mean education F, 15-44 (Years)	8.45	3.40	0.7 to 15.07	Interval	Mean years of education achieved by women aged 15-44. Captures the effect of youth and young adult education level on tobacco consumption.	IHME[11]

Appendix Table 10. Goodness of fit statistics for country-dummy, UN subregion, UN region, uninteracted, 2-year distributed lag, and two-year distributed lag event models. Root mean squared error (RMSE), mean absolute error (MAE), mean absolute percent error (MAPE), and Theil's U presented from top to bottom.

	Country-dummy model	UN subregion model	UN region model	Uninteracted model	1-year distributed lag model	2-year distributed lag model
RMSE	29.37	53.14	66.48	124.18	34.02	44.01
MAE	22.27	45.09	48.70	96.96	25.65	33.85
MAPE	0.02	0.03	0.03	0.07	0.02	0.02
Theil's U	1.05	1.86	2.51	4.33	1.24	1.59

Appendix Figure 1. Proportion of the world's population and the verified sample population covered by data from 1970-2013. Over 85% of the world's population and over 94% of the sample is covered by data over the entire sample period.

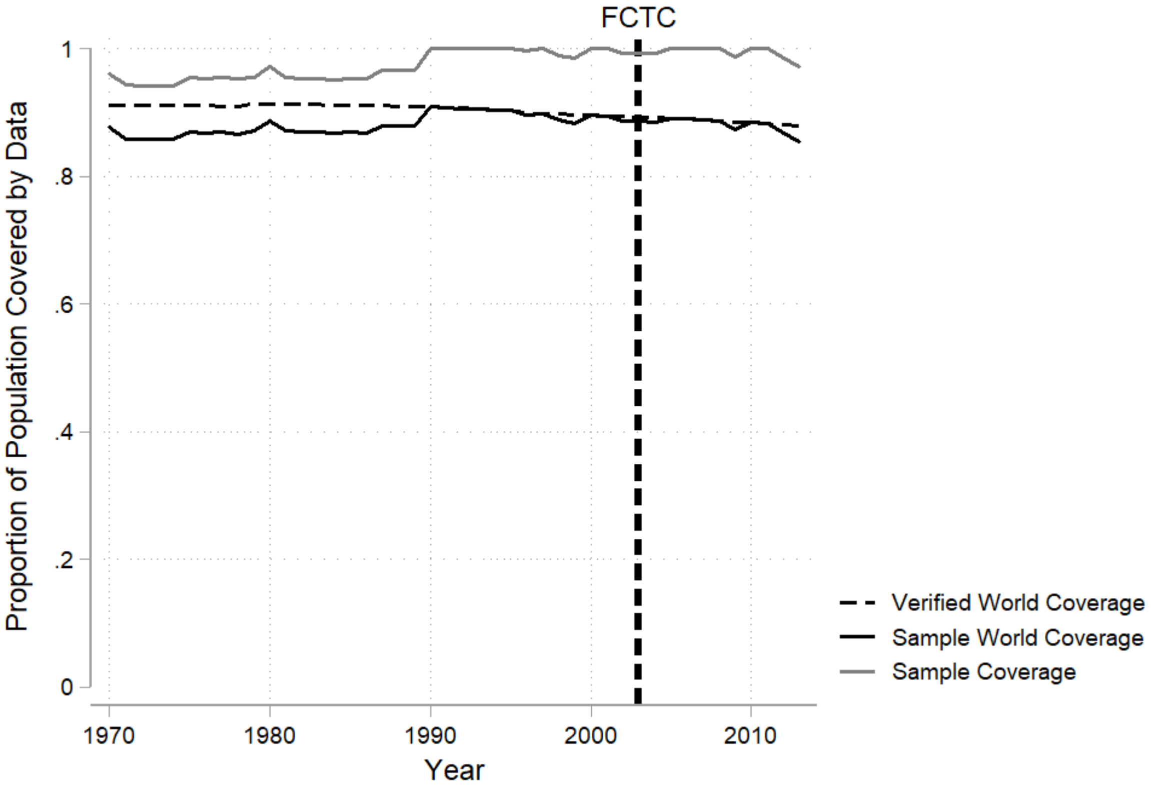

Appendix Figure 2. Annual population-weighted cigarette consumption per person aligned by the actual year that each country signed the FCTC and the year that each country ratified the FCTC, with year 0 as year of intervention. Actual year-aligned data points are different than Figure 1 because only countries that have signed and ratified the FCTC are included for analysis.

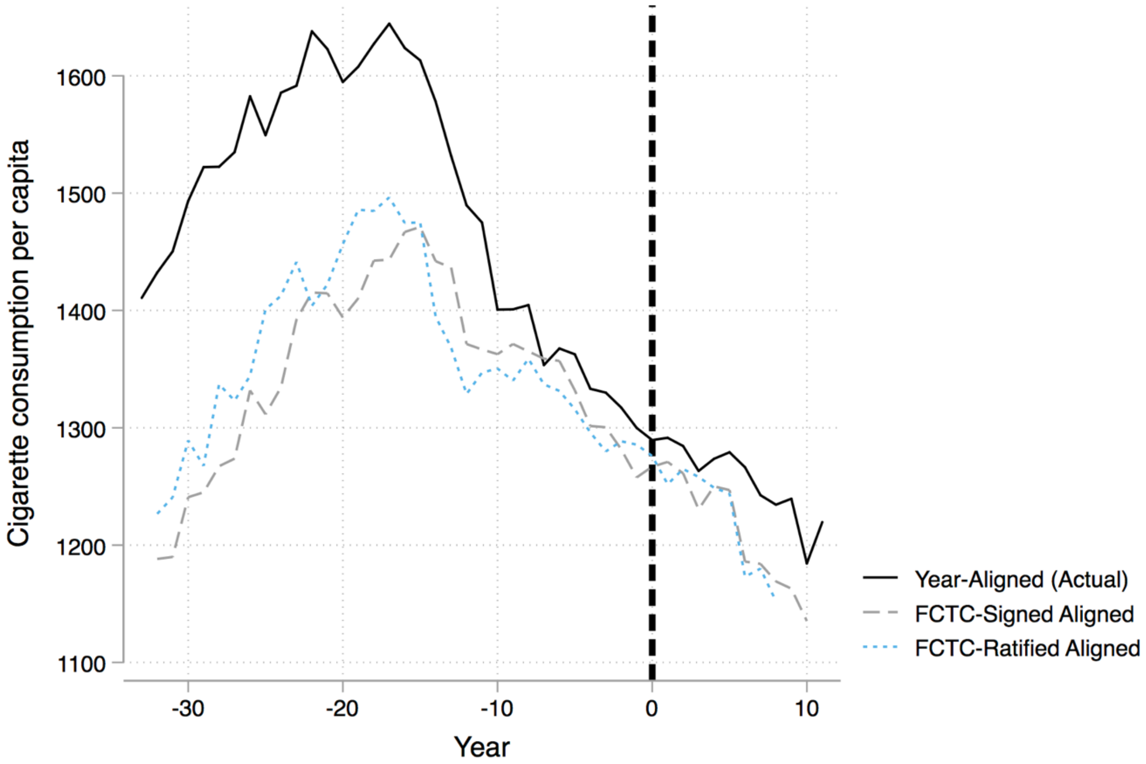

Appendix to Hoffman SJ et al. 2019. BMJ. doi: 10.1136/bmj.l2287

Appendix Figure 3. Interrupted time series plot of annual change in (first-differenced) global population-weighted cigarette consumption per capita with country-specific FCTC signing year as the intervention year and 95% confidence intervals for both pre- and post-FCTC trendlines.

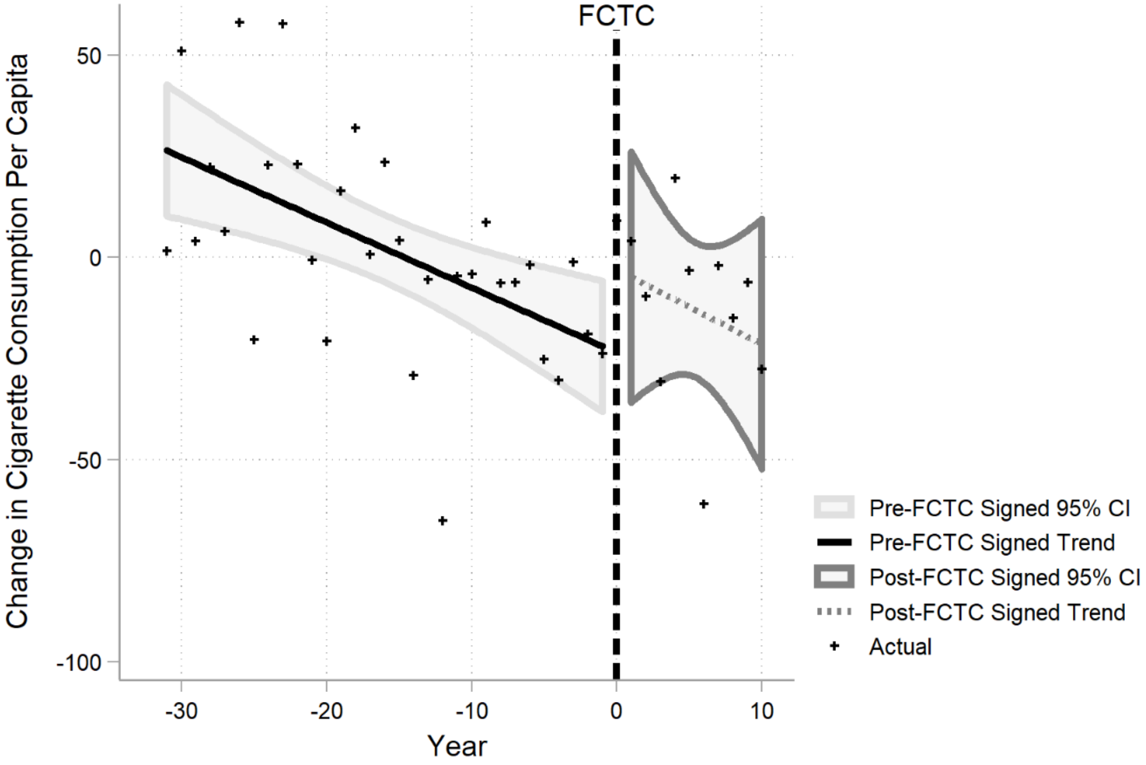

Appendix to Hoffman SJ et al. 2019. BMJ. doi: 10.1136/bmj.l2287

Appendix Figure 4. Interrupted time series plot of annual change in (first-differenced) global population-weighted cigarette consumption per capita with country-specific FCTC ratification year as the intervention year and 95% confidence intervals for both pre- and post-FCTC trendlines.

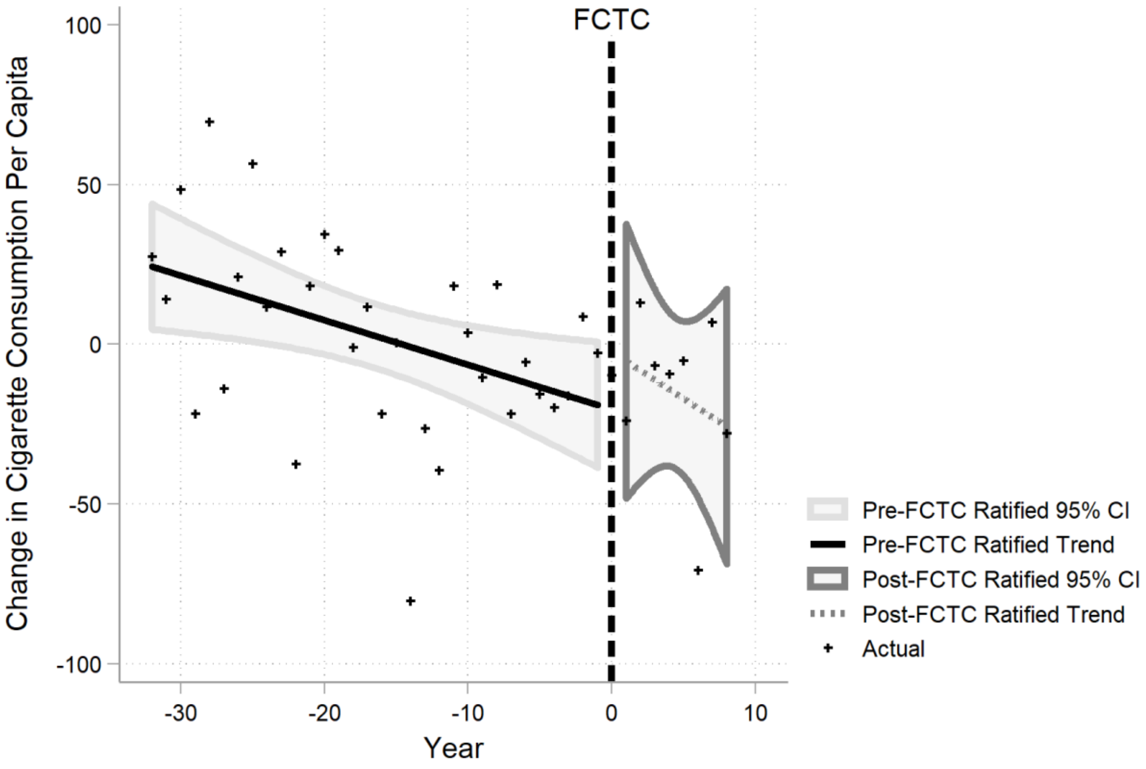

Appendix to Hoffman SJ et al. 2019. BMJ. doi: 10.1136/bmj.l2287

Appendix Figure 5. Interrupted time series plot of annual change in (first-differenced) global population-weighted cigarette consumption per capita with 1999 intervention year and 95% confidence intervals for both pre- and post-FCTC trendlines.

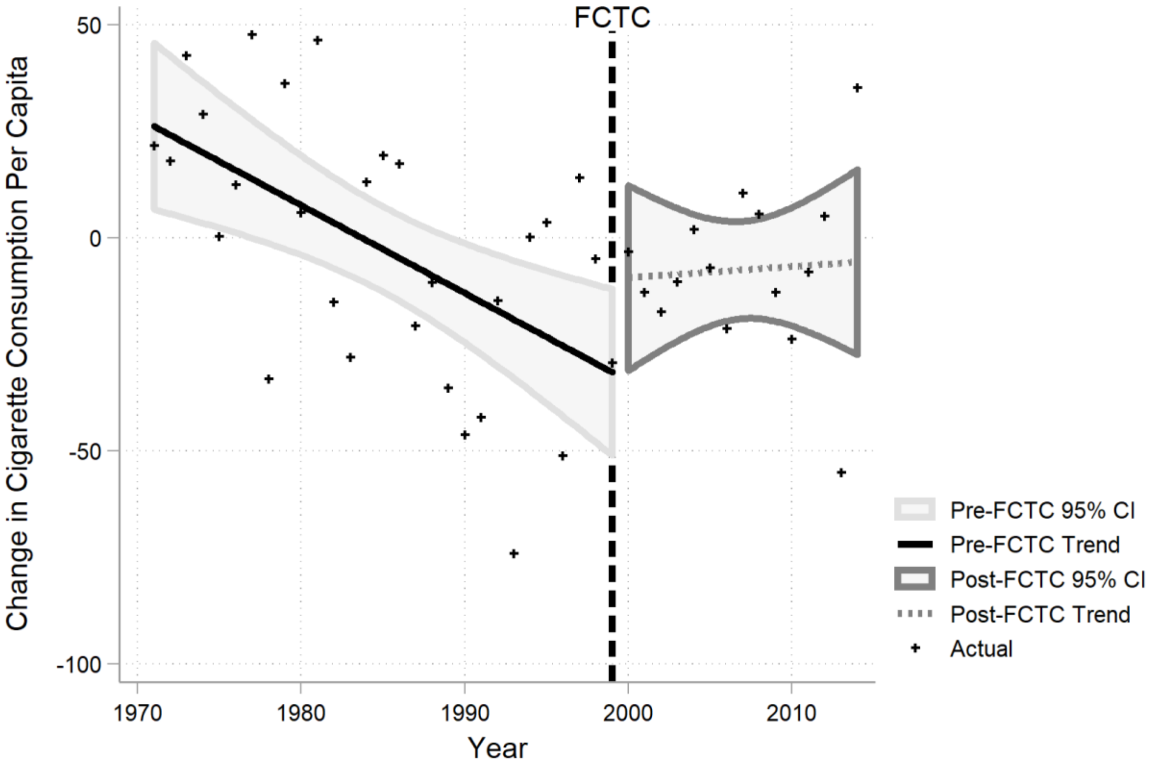

Appendix to Hoffman SJ et al. 2019. BMJ. doi: 10.1136/bmj.l2287

Appendix Figure 6. Country-dummy event model prediction of cigarette consumption per capita for the top 10 cigarette consuming countries, including 95% prediction intervals. In-sample forecast cutoff begins in 2003, after which predictions are based on economic, democracy, education, gender equity, tobacco industry, year, and country-dummy coefficients.

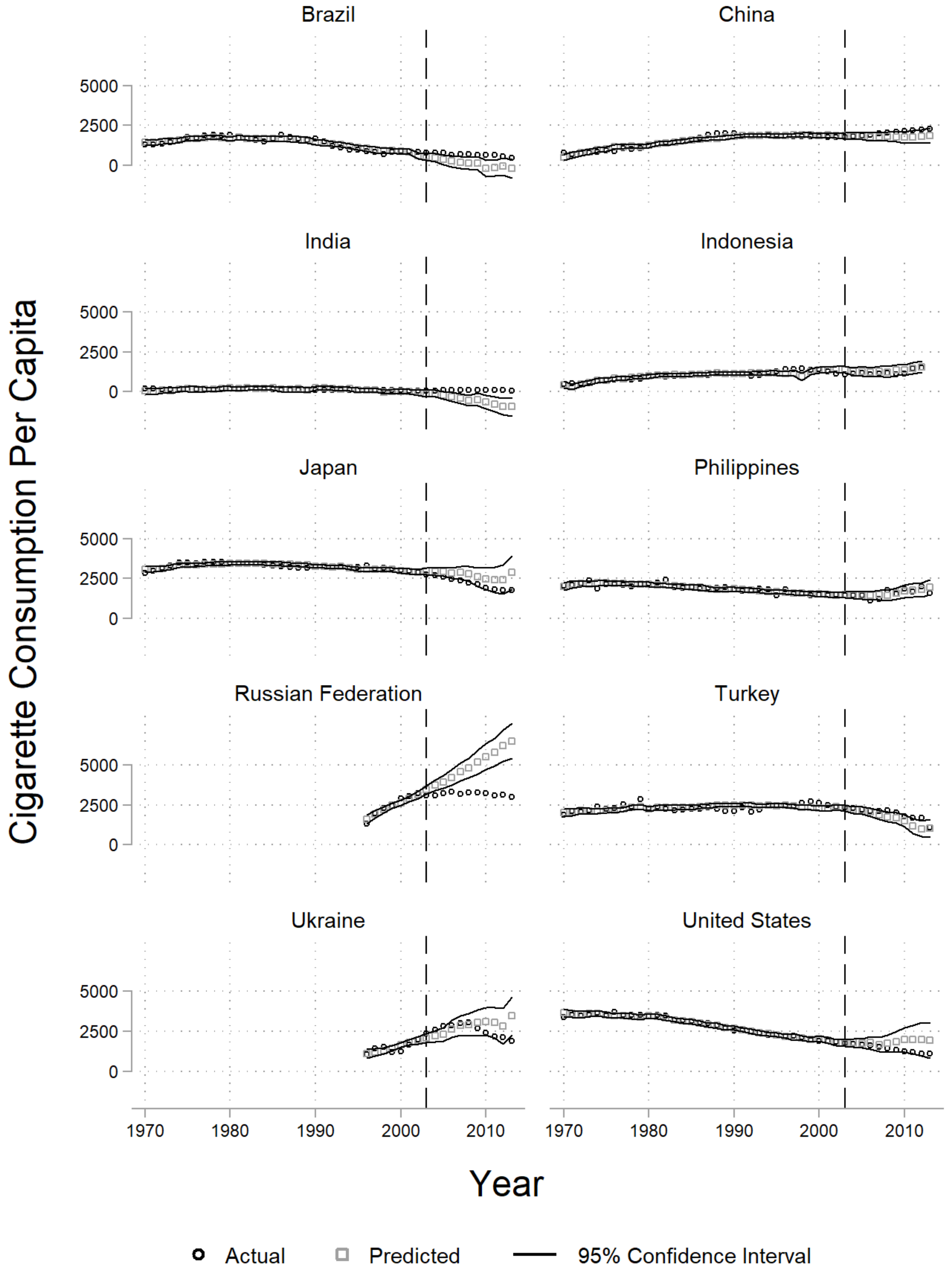

Appendix to Hoffman SJ et al. 2019. BMJ. doi: 10.1136/bmj.l2287

Appendix Figure 7. Population-weighted global event model prediction of cigarette consumption per capita, including 95% prediction intervals comparing primary model with a model based on only one measure of GDP. In-sample forecast cutoff begins in 2003, after which predictions are based on economic, democracy, education, gender equity, tobacco industry, year, and country-dummy coefficients.

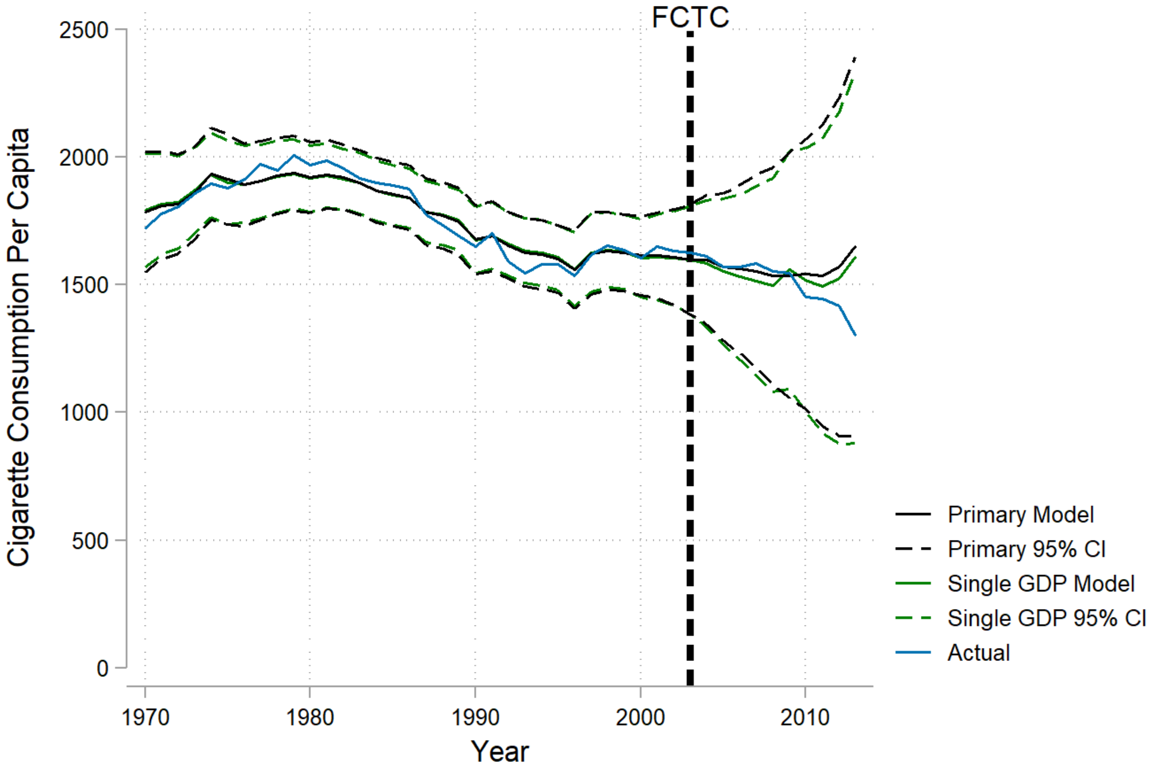

Appendix to Hoffman SJ et al. 2019. BMJ. doi: 10.1136/bmj.l2287

Appendix Figure 8. Population-weighted global event model prediction of cigarette consumption per capita for the Americas, split up by US and Canada (left) and Latin America (right) including 80%, 90%, and 95% prediction intervals. In-sample forecast cutoff begins in 2003, after which predictions are based on economic, democracy, education, gender equity, tobacco industry, year, and country-dummy coefficients.

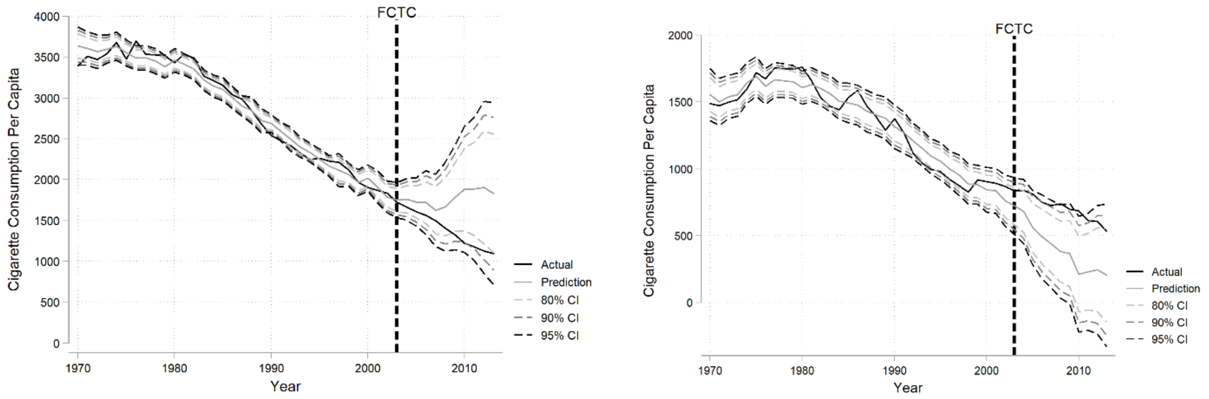

Appendix to Hoffman SJ et al. 2019. BMJ. doi: 10.1136/bmj.l2287

Appendix Figure 9. Population-weighted global event model prediction of cigarette consumption per capita for Africa including 80%, 90%, and 95% prediction intervals. In-sample forecast cutoff begins in 2003, after which predictions are based on economic, democracy, education, gender equity, tobacco industry, year, and country-dummy coefficients.

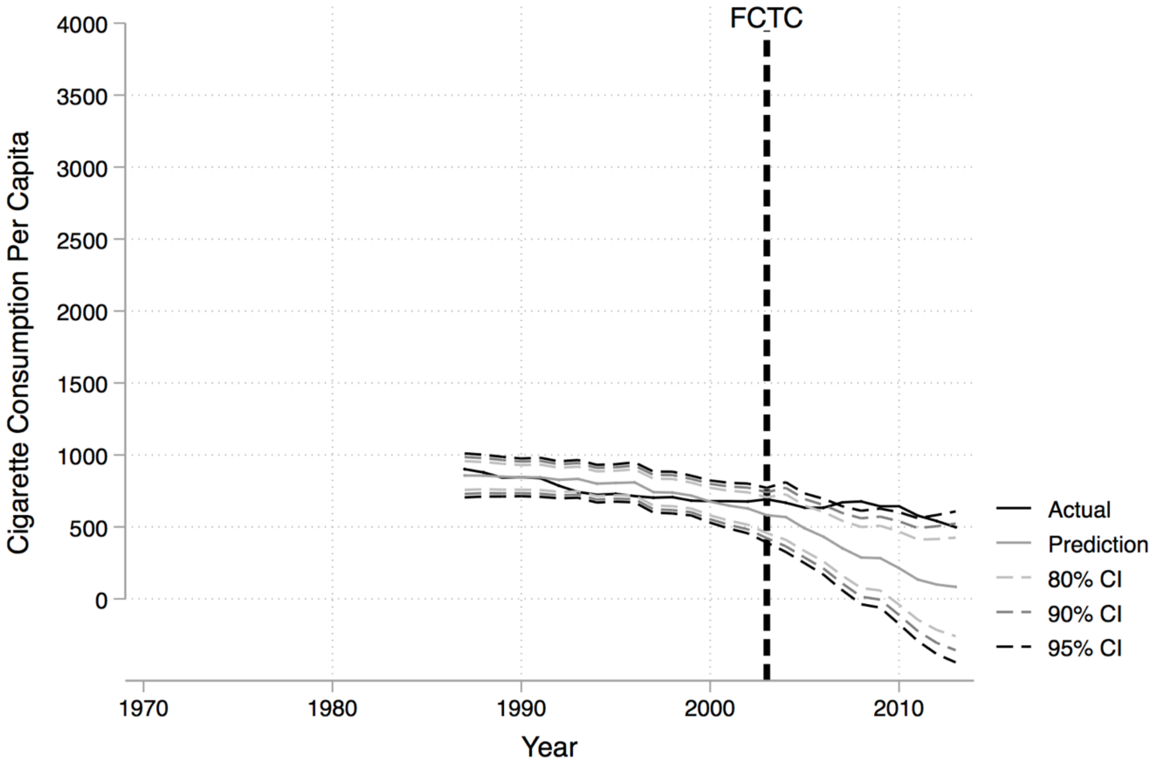

Appendix to Hoffman SJ et al. 2019. BMJ. doi: 10.1136/bmj.l2287

Appendix Figure 10. Global event model prediction of cigarette consumption per capita with a one-year distributed lag of explanatory variables, including 95% prediction intervals. In-sample forecast cutoff begins in 2003, after which predictions are based on economy, political system, tobacco industry, and human development coefficients for the current and previous year.

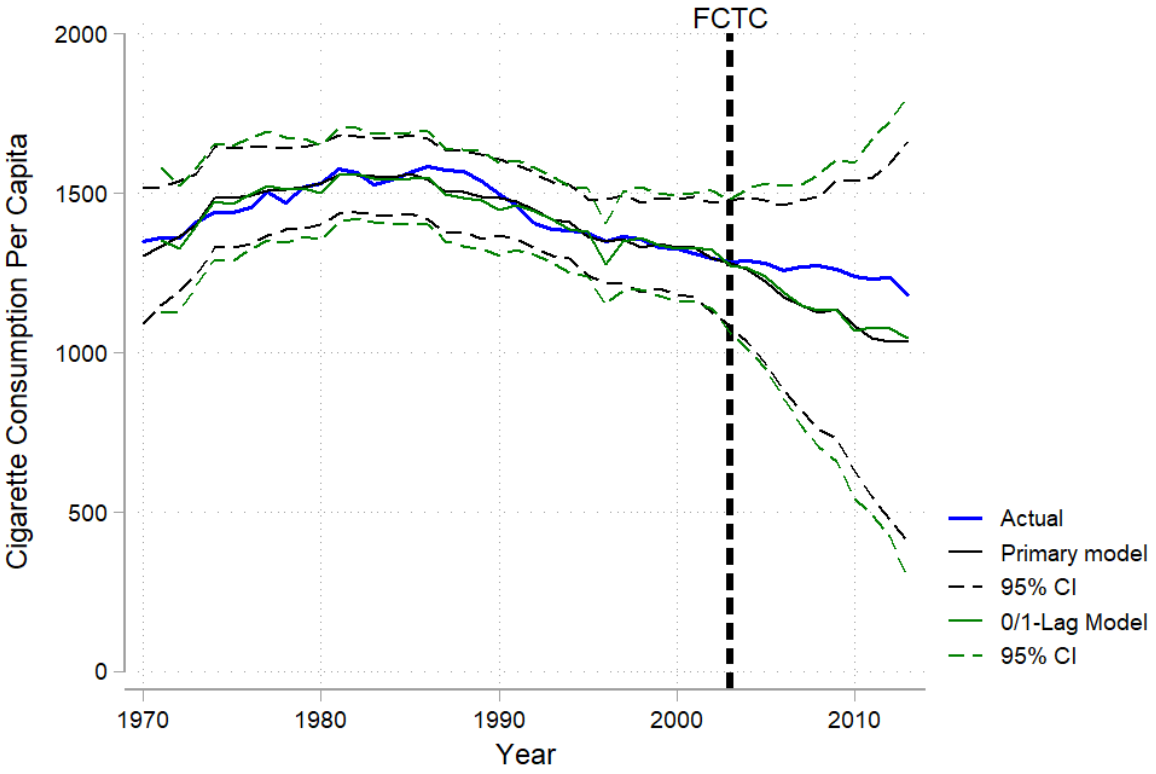

Appendix Figure 11. Global event model prediction of cigarette consumption per capita with a two-year distributed lag of explanatory variables, including 95% prediction intervals. In-sample forecast cutoff begins in 2003, after which predictions are based on economy, political system, tobacco industry, and human development coefficients for the current and previous two years.

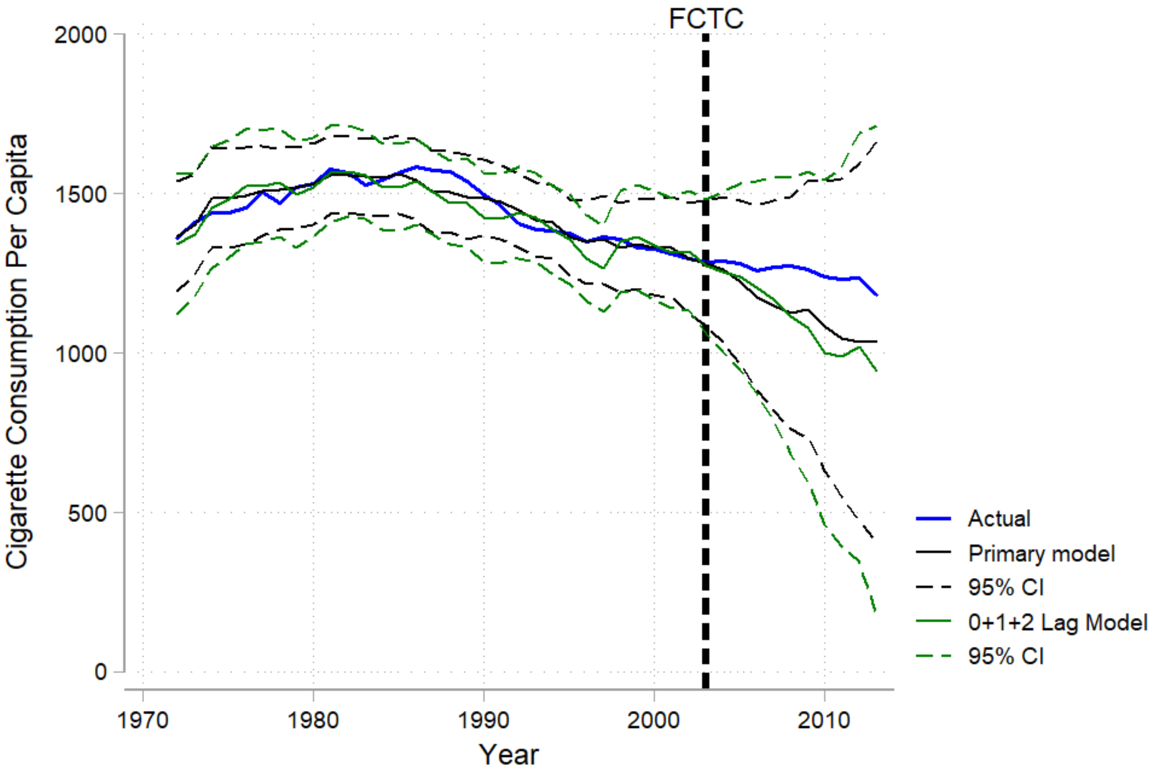

Appendix to Hoffman SJ et al. 2019. BMJ. doi: 10.1136/bmj.l2287

Appendix Figure 12. Population-weighted global event model prediction of cigarette consumption per capita excluding China, and including 80%, 90%, and 95% prediction intervals. In-sample forecast sample cutoff begins in 2003, after which predictions are based on economic, democracy, education, gender equity, tobacco industry, year, and country-dummy coefficients.

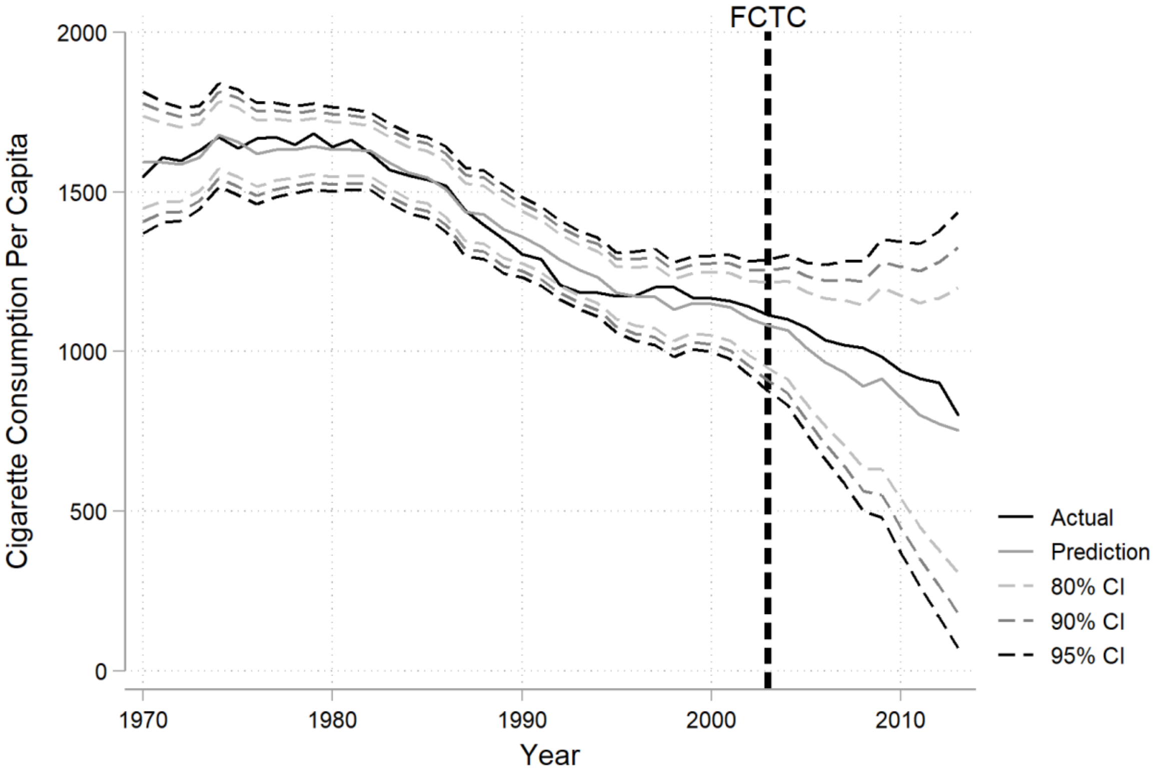

Appendix to Hoffman SJ et al. 2019. BMJ. doi: 10.1136/bmj.l2287

Appendix Figure 13. Unweighted global event model prediction of cigarette consumption per capita, including 80%, 90%, and 95% prediction intervals. In-sample forecast cutoff begins in 2003, after which predictions are based on economy, political system, tobacco industry, and human development coefficients.

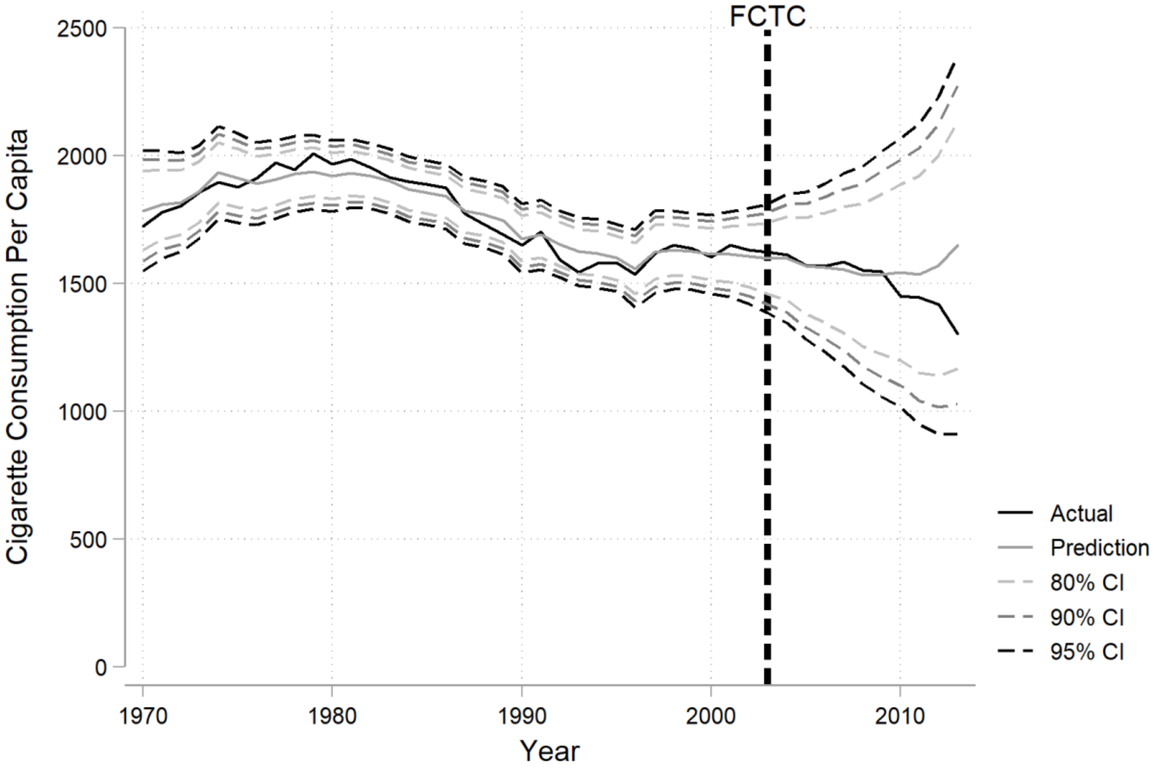

Appendix to Hoffman SJ et al. 2019. BMJ. doi: 10.1136/bmj.l2287
